# Error-threshold exists in fitness landscapes with lethal mutants

**DOI:** 10.1186/1471-2148-7-15

**Published:** 2007-02-07

**Authors:** Nobuto Takeuchi, Paulien Hogeweg

**Affiliations:** 1 Theoretical Biology/Bioinformatics Group, Utrecht University, Padualaan 8, 3584 CH Utrecht, The Netherlands

## Abstract

**Background:**

One of the important insights of quasi-species theory is an error-threshold. The error-threshold is the error rate of replication above which the sudden onset of the population delocalization from the fittest genotype occurs despite Darwinian selection; i.e., the break down of evolutionary optimization. However, a recent article by Wilke in this journal, after reviewing the previous studies on the error-threshold, concluded that the error-threshold does not exist if lethal mutants are taken into account in a fitness landscape. Since lethal mutants obviously exist in reality, this has a significant implication about biological evolution. However, the study of Wagner and Krall on which Wilke's conclusion was based considered mutation-selection dynamics in one-dimensional genotype space with the assumption that a genotype can mutate only to an adjoining genotype in the genotype space. In this article, we study whether the above conclusion holds in high-dimensional genotype space without the assumption of the adjacency of mutations, where the consequences of mutation-selection dynamics can be qualitatively different.

**Results:**

To examine the effect of mutant lethality on the existence of the error-threshold, we extend the quasi-species equation by taking the lethality of mutants into account, assuming that lethal genotypes are uniformly distributed in the genotype space. First, with the simplification of neglecting back mutations, we calculate the error-threshold as the maximum allowable mutation rate for which the fittest genotype can survive. Second, with the full consideration of back mutations, we study the equilibrium population distribution and the ancestor distribution in the genotype space as a function of error rate with and without lethality in a multiplicative fitness landscape. The results show that a high lethality of mutants actually introduces an error-threshold in a multiplicative fitness landscape in sharp contrast to the conclusion of Wilke. Furthermore, irrespective of the lethality of mutants, the delocalization of the population from the fittest genotype occurs for an error rate much smaller than random replication. Finally, the results are shown to extend to a system of finite populations.

**Conclusion:**

High lethality of mutants introduces an error-threshold in a multiplicative fitness landscape. Furthermore, irrespective of the lethality of mutants, the break down of evolutionary optimization happens for an error rate much smaller than random replication.

## Background

One of the important insights of quasi-species theory is the existence of an error-threshold [1-3]. The error-threshold is the error rate of replication above which the sudden onset of the population delocalization from the fittest genotype occurs despite Darwinian selection; i.e., the break down of evolutionary optimization. The error-threshold hampers the evolutionary accumulation of information through increasing the genome size of the replicator because a longer genome receives mutations more frequently for a given error rate [1,4].

A recent article of Wilke reviewed the quasi-species theory in the context of population genetics [5]. While the use of quasi-species theory in understanding of virus evolution is often seen (e.g. [6]), some studies argue that quasi-species theory contradicts population genetics, questioning the pertinence of quasi-species theory in this context (e.g. [7]). The article by Wilke resolved such misconceptions, and pointed out the usefulness and the problems of the current theory of virus evolution.

However, Wilke also argued that the existence of an error-threshold is one of the "beliefs" about quasi-species theory [5]. To quote: "*Can the error threshold occur in a more realistic fitness landscape that contains lethal genotypes? No. Wagner and Krall have proven mathematically that the condition for the existence of an error threshold is precisely the complete absence of lethal genotypes *[8]". Since lethal mutants obviously exist, the above statement has a significant implication about the relevance of the error-threshold in biological evolution: in particular, the accumulation of information. However, the study of Wagner and Krall [8], on which his conclusion was based, considered mutation-selection dynamics in one-dimensional genotype space (a chain of genotypes) with the assumption that a genotype can mutate only to another genotype adjacent to the original one in the genotype space. In contrast, quasi-species theory explicitly considers high-dimensional genotype space (i.e., sequence space) with the consideration of both adjacent and non-adjacent mutations. Since the possibility of mutations can be considerably greater in the latter setting, this can give rise to qualitative differences in the mutation-selection dynamics.

Furthermore, high-dimensional sequence space is a more realistic (and yet simple) representation of genotype space than one-dimensional genotype chain in view of a genome as sequences of DNA (or RNA in some cases). Thus, the aim of this article is to examine the above statement about the effect of lethality on the error-threshold by considering high-dimensional genotype space with the inclusion of non-adjacent mutations.

## Results

### Model

Before considering the lethality of mutants, let us introduce the quasi-species equation by following [2]. Let a genotype be a sequence of 0's and 1's (a bit sequence) of a fixed length *ν *– then, all possible genotypes form a *ν*-dimensional sequence space. Let a certain genotype G
 MathType@MTEF@5@5@+=feaafiart1ev1aaatCvAUfKttLearuWrP9MDH5MBPbIqV92AaeXatLxBI9gBaebbnrfifHhDYfgasaacH8akY=wiFfYdH8Gipec8Eeeu0xXdbba9frFj0=OqFfea0dXdd9vqai=hGuQ8kuc9pgc9s8qqaq=dirpe0xb9q8qiLsFr0=vr0=vr0dc8meaabaqaciaacaGaaeqabaqabeGadaaakeaat0uy0HwzTfgDPnwy1egaryqtHrhAL1wy0L2yHvdaiqaacqWFge=raaa@382C@_0 _have the greatest reproduction rate. Let us assume that the reproduction rate of mutants is a decreasing function of the number of point mutations (i.e., substitutions) from G
 MathType@MTEF@5@5@+=feaafiart1ev1aaatCvAUfKttLearuWrP9MDH5MBPbIqV92AaeXatLxBI9gBaebbnrfifHhDYfgasaacH8akY=wiFfYdH8Gipec8Eeeu0xXdbba9frFj0=OqFfea0dXdd9vqai=hGuQ8kuc9pgc9s8qqaq=dirpe0xb9q8qiLsFr0=vr0=vr0dc8meaabaqaciaacaGaaeqabaqabeGadaaakeaat0uy0HwzTfgDPnwy1egaryqtHrhAL1wy0L2yHvdaiqaacqWFge=raaa@382C@_0_. Let us denote the class of genotypes carrying *d *substitutions by G
 MathType@MTEF@5@5@+=feaafiart1ev1aaatCvAUfKttLearuWrP9MDH5MBPbIqV92AaeXatLxBI9gBaebbnrfifHhDYfgasaacH8akY=wiFfYdH8Gipec8Eeeu0xXdbba9frFj0=OqFfea0dXdd9vqai=hGuQ8kuc9pgc9s8qqaq=dirpe0xb9q8qiLsFr0=vr0=vr0dc8meaabaqaciaacaGaaeqabaqabeGadaaakeaat0uy0HwzTfgDPnwy1egaryqtHrhAL1wy0L2yHvdaiqaacqWFge=raaa@382C@_*d*_, the reproduction rate of G
 MathType@MTEF@5@5@+=feaafiart1ev1aaatCvAUfKttLearuWrP9MDH5MBPbIqV92AaeXatLxBI9gBaebbnrfifHhDYfgasaacH8akY=wiFfYdH8Gipec8Eeeu0xXdbba9frFj0=OqFfea0dXdd9vqai=hGuQ8kuc9pgc9s8qqaq=dirpe0xb9q8qiLsFr0=vr0=vr0dc8meaabaqaciaacaGaaeqabaqabeGadaaakeaat0uy0HwzTfgDPnwy1egaryqtHrhAL1wy0L2yHvdaiqaacqWFge=raaa@382C@_*d *_by *f*_*d*_, the population fraction of G
 MathType@MTEF@5@5@+=feaafiart1ev1aaatCvAUfKttLearuWrP9MDH5MBPbIqV92AaeXatLxBI9gBaebbnrfifHhDYfgasaacH8akY=wiFfYdH8Gipec8Eeeu0xXdbba9frFj0=OqFfea0dXdd9vqai=hGuQ8kuc9pgc9s8qqaq=dirpe0xb9q8qiLsFr0=vr0=vr0dc8meaabaqaciaacaGaaeqabaqabeGadaaakeaat0uy0HwzTfgDPnwy1egaryqtHrhAL1wy0L2yHvdaiqaacqWFge=raaa@382C@_*d *_by *y*_*d *_and the probability of mutation from G
 MathType@MTEF@5@5@+=feaafiart1ev1aaatCvAUfKttLearuWrP9MDH5MBPbIqV92AaeXatLxBI9gBaebbnrfifHhDYfgasaacH8akY=wiFfYdH8Gipec8Eeeu0xXdbba9frFj0=OqFfea0dXdd9vqai=hGuQ8kuc9pgc9s8qqaq=dirpe0xb9q8qiLsFr0=vr0=vr0dc8meaabaqaciaacaGaaeqabaqabeGadaaakeaat0uy0HwzTfgDPnwy1egaryqtHrhAL1wy0L2yHvdaiqaacqWFge=raaa@382C@_*d *_to G
 MathType@MTEF@5@5@+=feaafiart1ev1aaatCvAUfKttLearuWrP9MDH5MBPbIqV92AaeXatLxBI9gBaebbnrfifHhDYfgasaacH8akY=wiFfYdH8Gipec8Eeeu0xXdbba9frFj0=OqFfea0dXdd9vqai=hGuQ8kuc9pgc9s8qqaq=dirpe0xb9q8qiLsFr0=vr0=vr0dc8meaabaqaciaacaGaaeqabaqabeGadaaakeaat0uy0HwzTfgDPnwy1egaryqtHrhAL1wy0L2yHvdaiqaacqWFge=raaa@382C@_*e *_per replication by *m*_*ed*_. In the case of *e *= *d*, *m*_*ed *_is reformulated as the probability of correct replication, plus the mutation probability from one genotype in G
 MathType@MTEF@5@5@+=feaafiart1ev1aaatCvAUfKttLearuWrP9MDH5MBPbIqV92AaeXatLxBI9gBaebbnrfifHhDYfgasaacH8akY=wiFfYdH8Gipec8Eeeu0xXdbba9frFj0=OqFfea0dXdd9vqai=hGuQ8kuc9pgc9s8qqaq=dirpe0xb9q8qiLsFr0=vr0=vr0dc8meaabaqaciaacaGaaeqabaqabeGadaaakeaat0uy0HwzTfgDPnwy1egaryqtHrhAL1wy0L2yHvdaiqaacqWFge=raaa@382C@_*d *_to another genotype in G
 MathType@MTEF@5@5@+=feaafiart1ev1aaatCvAUfKttLearuWrP9MDH5MBPbIqV92AaeXatLxBI9gBaebbnrfifHhDYfgasaacH8akY=wiFfYdH8Gipec8Eeeu0xXdbba9frFj0=OqFfea0dXdd9vqai=hGuQ8kuc9pgc9s8qqaq=dirpe0xb9q8qiLsFr0=vr0=vr0dc8meaabaqaciaacaGaaeqabaqabeGadaaakeaat0uy0HwzTfgDPnwy1egaryqtHrhAL1wy0L2yHvdaiqaacqWFge=raaa@382C@_*d*_. Given a sufficiently large population size and asexual reproduction, the dynamics of *y*_*d *_follows

dyd/dt=fdmddyd+∑e≠dfemdeye−yd∑i=0νfiyi.     (1)
 MathType@MTEF@5@5@+=feaafiart1ev1aaatCvAUfKttLearuWrP9MDH5MBPbIqV92AaeXatLxBI9gBaebbnrfifHhDYfgasaacH8akY=wiFfYdH8Gipec8Eeeu0xXdbba9frFj0=OqFfea0dXdd9vqai=hGuQ8kuc9pgc9s8qqaq=dirpe0xb9q8qiLsFr0=vr0=vr0dc8meaabaqaciaacaGaaeqabaqabeGadaaakeaacqWGKbazcqWG5bqEdaWgaaWcbaGaemizaqgabeaakiabc+caViabdsgaKjabdsha0jabg2da9iabdAgaMnaaBaaaleaacqWGKbazaeqaaOGaemyBa02aaSbaaSqaaiabdsgaKjabdsgaKbqabaGccqWG5bqEdaWgaaWcbaGaemizaqgabeaakiabgUcaRmaaqafabaGaemOzay2aaSbaaSqaaiabdwgaLbqabaGccqWGTbqBdaWgaaWcbaGaemizaqMaemyzaugabeaakiabdMha5naaBaaaleaacqWGLbqzaeqaaaqaaiabdwgaLjabgcMi5kabdsgaKbqab0GaeyyeIuoakiabgkHiTiabdMha5naaBaaaleaacqWGKbazaeqaaOWaaabCaeaacqWGMbGzdaWgaaWcbaGaemyAaKgabeaakiabdMha5naaBaaaleaacqWGPbqAaeqaaaqaaiabdMgaPjabg2da9iabicdaWaqaaGGaciab=17aUbqdcqGHris5aOGaeiOla4IaaCzcaiaaxMaadaqadaqaaiabigdaXaGaayjkaiaawMcaaaaa@6711@

In the RHS of Eq. (1), the first term is the reproduction of G
 MathType@MTEF@5@5@+=feaafiart1ev1aaatCvAUfKttLearuWrP9MDH5MBPbIqV92AaeXatLxBI9gBaebbnrfifHhDYfgasaacH8akY=wiFfYdH8Gipec8Eeeu0xXdbba9frFj0=OqFfea0dXdd9vqai=hGuQ8kuc9pgc9s8qqaq=dirpe0xb9q8qiLsFr0=vr0=vr0dc8meaabaqaciaacaGaaeqabaqabeGadaaakeaat0uy0HwzTfgDPnwy1egaryqtHrhAL1wy0L2yHvdaiqaacqWFge=raaa@382C@_*d*_, the second term is the mutation fluxes from all other genotype classes; the last term keeps the total population size constant. Let us calculate *m*_*de *_by considering only point mutations. Assuming that the probability of correct replication per character per replication – let us denote this by *q *– is uniform along the sequence location and constant among genotypes, the mutation probability *m*_*de *_is

mde=qν∑i=0c(1/q−1)2i+|e−d|(ν−ei+(|e−d|−e+d)/2)(ei+(|e−d|+e−d)/2),
 MathType@MTEF@5@5@+=feaafiart1ev1aaatCvAUfKttLearuWrP9MDH5MBPbIqV92AaeXatLxBI9gBaebbnrfifHhDYfgasaacH8akY=wiFfYdH8Gipec8Eeeu0xXdbba9frFj0=OqFfea0dXdd9vqai=hGuQ8kuc9pgc9s8qqaq=dirpe0xb9q8qiLsFr0=vr0=vr0dc8meaabaqaciaacaGaaeqabaqabeGadaaakeaafaqaaeGabaaabaGaemyBa02aaSbaaSqaaiabdsgaKjabdwgaLbqabaGccqGH9aqpcqWGXbqCdaahaaWcbeqaaGGaciab=17aUbaakmaaqahabaGaeiikaGIaeGymaeJaei4la8IaemyCaeNaeyOeI0IaeGymaeJaeiykaKYaaWbaaSqabeaacqaIYaGmcqWGPbqAcqGHRaWkdaabdaqaaiabdwgaLjabgkHiTiabdsgaKbGaay5bSlaawIa7aaaaaeaacqWGPbqAcqGH9aqpcqaIWaamaeaacqWGJbWya0GaeyyeIuoaaOqaamaabmaabaqbaeqabiqaaaqaaiab=17aUjabgkHiTiabdwgaLbqaaiabdMgaPjabgUcaRmaabmaabaWaaqWaaeaacqWGLbqzcqGHsislcqWGKbazaiaawEa7caGLiWoacqGHsislcqWGLbqzcqGHRaWkcqWGKbazaiaawIcacaGLPaaacqGGVaWlcqaIYaGmaaaacaGLOaGaayzkaaWaaeWaaeaafaqabeGabaaabaGaemyzaugabaGaemyAaKMaey4kaSYaaeWaaeaadaabdaqaaiabdwgaLjabgkHiTiabdsgaKbGaay5bSlaawIa7aiabgUcaRiabdwgaLjabgkHiTiabdsgaKbGaayjkaiaawMcaaiabc+caViabikdaYaaaaiaawIcacaGLPaaacqGGSaalaaaaaa@77F4@

where *c *= {min(*e *+ *d*, 2*ν *- *e *- *d*) - |*e *- *d*|}/2 (see [2]). Note that a mutation in the current model can be composed of multiple substitutions (non-adjacent mutation) because the average number of substitutions per replication, (1 - *q*)*ν *(commonly denoted by *U *in the population genetics theory), can be greater than 1. Below, we introduce the lethality of mutants in Eq. (1). A lethal mutant is here defined as a mutant which dies no later than it is born. If epistatic interactions between sites in a genome are completely absent in terms of the lethality, some sites can be fixed as lethal sites, and the others as non-lethal sites. By excluding these lethal sites from the consideration, the model can be treated as if there are no lethal mutants. Models without lethal mutants have been already studied. For example, it was shown in [9] that, for multiplicative fitness landscape, the population is delocalized without an error-threshold (without any sudden transition-like behavior) as the mutation rate increases. Instead, we here consider the case of fully epistatic interaction by assuming that the non-lethal mutants are uniformly distributed in the genotype space. Let *κ *denote the fraction of non-lethal mutants in the genotype space. Then the lethality is defined as 1 - *κ*, which takes a value between 0 and 1. Then, Eq. (1) becomes

dyd/dt=fd(qν+κ˜d(mdd−qν))yd+∑e≠dfeκmdeye−ydΦ,     (2)
 MathType@MTEF@5@5@+=feaafiart1ev1aaatCvAUfKttLearuWrP9MDH5MBPbIqV92AaeXatLxBI9gBaebbnrfifHhDYfgasaacH8akY=wiFfYdH8Gipec8Eeeu0xXdbba9frFj0=OqFfea0dXdd9vqai=hGuQ8kuc9pgc9s8qqaq=dirpe0xb9q8qiLsFr0=vr0=vr0dc8meaabaqaciaacaGaaeqabaqabeGadaaakeaafaqadeGabaaabaGaemizaqMaemyEaK3aaSbaaSqaaiabdsgaKbqabaGccqGGVaWlcqWGKbazcqWG0baDcqGH9aqpcqWGMbGzdaWgaaWcbaGaemizaqgabeaakmaabmaabaGaemyCae3aaWbaaSqabeaaiiGacqWF9oGBaaGccqGHRaWkcuWF6oWAgaacamaaBaaaleaacqWGKbazaeqaaOWaaeWaaeaacqWGTbqBdaWgaaWcbaGaemizaqMaemizaqgabeaakiabgkHiTiabdghaXnaaCaaaleqabaGae8xVd4gaaaGccaGLOaGaayzkaaaacaGLOaGaayzkaaGaemyEaK3aaSbaaSqaaiabdsgaKbqabaaakeaacqGHRaWkdaaeqbqaaiabdAgaMnaaBaaaleaacqWGLbqzaeqaaOGae8NUdSMaemyBa02aaSbaaSqaaiabdsgaKjabdwgaLbqabaGccqWG5bqEdaWgaaWcbaGaemyzaugabeaakiabgkHiTiabdMha5naaBaaaleaacqWGKbazaeqaaOGaeuOPdyKaeiilaWcaleaacqWGLbqzcqGHGjsUcqWGKbazaeqaniabggHiLdaaaOGaaCzcaiaaxMaadaqadaqaaiabikdaYaGaayjkaiaawMcaaaaa@6BFD@

where κ˜
 MathType@MTEF@5@5@+=feaafiart1ev1aaatCvAUfKttLearuWrP9MDH5MBPbIqV92AaeXatLxBI9gBaebbnrfifHhDYfgasaacH8akY=wiFfYdH8Gipec8Eeeu0xXdbba9frFj0=OqFfea0dXdd9vqai=hGuQ8kuc9pgc9s8qqaq=dirpe0xb9q8qiLsFr0=vr0=vr0dc8meaabaqaciaacaGaaeqabaqabeGadaaakeaaiiGacuWF6oWAgaacaaaa@2E74@_*d *_denotes (k(νd)−1)/((νd)−1)
 MathType@MTEF@5@5@+=feaafiart1ev1aaatCvAUfKttLearuWrP9MDH5MBPbIqV92AaeXatLxBI9gBaebbnrfifHhDYfgasaacH8akY=wiFfYdH8Gipec8Eeeu0xXdbba9frFj0=OqFfea0dXdd9vqai=hGuQ8kuc9pgc9s8qqaq=dirpe0xb9q8qiLsFr0=vr0=vr0dc8meaabaqaciaacaGaaeqabaqabeGadaaakeaadaWcgaqaamaabmaabaGaem4AaS2aaeWaaeaafaqabeGabaaabaacciGae8xVd4gabaGaemizaqgaaaGaayjkaiaawMcaaiabgkHiTiabigdaXaGaayjkaiaawMcaaaqaamaabmaabaWaaeWaaeaafaqabeGabaaabaGae8xVd4gabaGaemizaqgaaaGaayjkaiaawMcaaiabgkHiTiabigdaXaGaayjkaiaawMcaaaaaaaa@3E2D@, which is the fraction of non-lethal mutants in G
 MathType@MTEF@5@5@+=feaafiart1ev1aaatCvAUfKttLearuWrP9MDH5MBPbIqV92AaeXatLxBI9gBaebbnrfifHhDYfgasaacH8akY=wiFfYdH8Gipec8Eeeu0xXdbba9frFj0=OqFfea0dXdd9vqai=hGuQ8kuc9pgc9s8qqaq=dirpe0xb9q8qiLsFr0=vr0=vr0dc8meaabaqaciaacaGaaeqabaqabeGadaaakeaat0uy0HwzTfgDPnwy1egaryqtHrhAL1wy0L2yHvdaiqaacqWFge=raaa@382C@_*d *_excluding one non-lethal mutant (but κ˜
 MathType@MTEF@5@5@+=feaafiart1ev1aaatCvAUfKttLearuWrP9MDH5MBPbIqV92AaeXatLxBI9gBaebbnrfifHhDYfgasaacH8akY=wiFfYdH8Gipec8Eeeu0xXdbba9frFj0=OqFfea0dXdd9vqai=hGuQ8kuc9pgc9s8qqaq=dirpe0xb9q8qiLsFr0=vr0=vr0dc8meaabaqaciaacaGaaeqabaqabeGadaaakeaaiiGacuWF6oWAgaacaaaa@2E74@_*d *= 0 _or *ν *= 0 by definition). The last term of Eq. (2) keeps the population size constant, and Φ is ∑i=0νfiyi(∑j≠iκmji+qν+κ˜i(mii−qν))
 MathType@MTEF@5@5@+=feaafiart1ev1aaatCvAUfKttLearuWrP9MDH5MBPbIqV92AaeXatLxBI9gBaebbnrfifHhDYfgasaacH8akY=wiFfYdH8Gipec8Eeeu0xXdbba9frFj0=OqFfea0dXdd9vqai=hGuQ8kuc9pgc9s8qqaq=dirpe0xb9q8qiLsFr0=vr0=vr0dc8meaabaqaciaacaGaaeqabaqabeGadaaakeaadaaeWaqaaiabdAgaMnaaBaaaleaacqWGPbqAaeqaaOGaemyEaK3aaSbaaSqaaiabdMgaPbqabaaabaGaemyAaKMaeyypa0JaeGimaadabaacciGae8xVd4ganiabggHiLdGcdaqadaqaamaaqababaGae8NUdSMaemyBa02aaSbaaSqaaiabdQgaQjabdMgaPbqabaGccqGHRaWkcqWGXbqCdaahaaWcbeqaaiab=17aUbaakiabgUcaRiqb=P7aRzaaiaWaaSbaaSqaaiabdMgaPbqabaGcdaqadaqaaiabd2gaTnaaBaaaleaacqWGPbqAcqWGPbqAaeqaaOGaeyOeI0IaemyCae3aaWbaaSqabeaacqWF9oGBaaaakiaawIcacaGLPaaaaSqaaiabdQgaQjabgcMi5kabdMgaPbqab0GaeyyeIuoaaOGaayjkaiaawMcaaaaa@5A16@. This term differs from the last term of Eq. (1) because lethal mutants do not contribute to the population in Eq. (2). This completes the formulation of the model.

The mathematical definition (detection) of error-threshold has been extensively discussed in literatures (e.g., see [10]). In the current study, we first adopt the same criterion as that employed in the study of Wagner and Krall [8], on which the conclusion of Wilke [5] is based, for comparison. In this criterion, by neglecting back mutations, the error-threshold can be calculated as the maximum allowable error rate for which the fittest genotype can survive [3,8,11,12]. Later, taking full consideration of back mutations, we study the effect of lethality on the error-threshold by examining the equilibrium population distribution and the ancestor distribution in the genotype space as a function of error rate.

### Error-threshold in the model without back mutations

In this section, we study the effect of lethality on the error-threshold by following the criterion of the error-threshold employed in [8]. In this criterion, the error-threshold is calculated as the error rate above which the fittest genotype cannot survive without back mutation. A back mutation is defined as a mutation which increases the fitness of a genotype as in [8]. For G
 MathType@MTEF@5@5@+=feaafiart1ev1aaatCvAUfKttLearuWrP9MDH5MBPbIqV92AaeXatLxBI9gBaebbnrfifHhDYfgasaacH8akY=wiFfYdH8Gipec8Eeeu0xXdbba9frFj0=OqFfea0dXdd9vqai=hGuQ8kuc9pgc9s8qqaq=dirpe0xb9q8qiLsFr0=vr0=vr0dc8meaabaqaciaacaGaaeqabaqabeGadaaakeaat0uy0HwzTfgDPnwy1egaryqtHrhAL1wy0L2yHvdaiqaacqWFge=raaa@382C@_0 _to survive without back mutations, G
 MathType@MTEF@5@5@+=feaafiart1ev1aaatCvAUfKttLearuWrP9MDH5MBPbIqV92AaeXatLxBI9gBaebbnrfifHhDYfgasaacH8akY=wiFfYdH8Gipec8Eeeu0xXdbba9frFj0=OqFfea0dXdd9vqai=hGuQ8kuc9pgc9s8qqaq=dirpe0xb9q8qiLsFr0=vr0=vr0dc8meaabaqaciaacaGaaeqabaqabeGadaaakeaat0uy0HwzTfgDPnwy1egaryqtHrhAL1wy0L2yHvdaiqaacqWFge=raaa@382C@_0 _must be able to invade at least G
 MathType@MTEF@5@5@+=feaafiart1ev1aaatCvAUfKttLearuWrP9MDH5MBPbIqV92AaeXatLxBI9gBaebbnrfifHhDYfgasaacH8akY=wiFfYdH8Gipec8Eeeu0xXdbba9frFj0=OqFfea0dXdd9vqai=hGuQ8kuc9pgc9s8qqaq=dirpe0xb9q8qiLsFr0=vr0=vr0dc8meaabaqaciaacaGaaeqabaqabeGadaaakeaat0uy0HwzTfgDPnwy1egaryqtHrhAL1wy0L2yHvdaiqaacqWFge=raaa@382C@_1_; i.e., the net reproduction rate of G
 MathType@MTEF@5@5@+=feaafiart1ev1aaatCvAUfKttLearuWrP9MDH5MBPbIqV92AaeXatLxBI9gBaebbnrfifHhDYfgasaacH8akY=wiFfYdH8Gipec8Eeeu0xXdbba9frFj0=OqFfea0dXdd9vqai=hGuQ8kuc9pgc9s8qqaq=dirpe0xb9q8qiLsFr0=vr0=vr0dc8meaabaqaciaacaGaaeqabaqabeGadaaakeaat0uy0HwzTfgDPnwy1egaryqtHrhAL1wy0L2yHvdaiqaacqWFge=raaa@382C@_0 _per individual must be greater than that of G
 MathType@MTEF@5@5@+=feaafiart1ev1aaatCvAUfKttLearuWrP9MDH5MBPbIqV92AaeXatLxBI9gBaebbnrfifHhDYfgasaacH8akY=wiFfYdH8Gipec8Eeeu0xXdbba9frFj0=OqFfea0dXdd9vqai=hGuQ8kuc9pgc9s8qqaq=dirpe0xb9q8qiLsFr0=vr0=vr0dc8meaabaqaciaacaGaaeqabaqabeGadaaakeaat0uy0HwzTfgDPnwy1egaryqtHrhAL1wy0L2yHvdaiqaacqWFge=raaa@382C@_1_. From Eq. (2), this condition reads *f*_0_*q*^*ν *^> *f*_1_*q*^*ν *^+ *f*_1_κ˜
 MathType@MTEF@5@5@+=feaafiart1ev1aaatCvAUfKttLearuWrP9MDH5MBPbIqV92AaeXatLxBI9gBaebbnrfifHhDYfgasaacH8akY=wiFfYdH8Gipec8Eeeu0xXdbba9frFj0=OqFfea0dXdd9vqai=hGuQ8kuc9pgc9s8qqaq=dirpe0xb9q8qiLsFr0=vr0=vr0dc8meaabaqaciaacaGaaeqabaqabeGadaaakeaaiiGacuWF6oWAgaacaaaa@2E74@_1_(*m*_11 _- *q*^*ν*^), and hence,

*f*_0_*q*^*ν *^> *f*_1_*q*^*ν *^+ *f*_1_κ˜
 MathType@MTEF@5@5@+=feaafiart1ev1aaatCvAUfKttLearuWrP9MDH5MBPbIqV92AaeXatLxBI9gBaebbnrfifHhDYfgasaacH8akY=wiFfYdH8Gipec8Eeeu0xXdbba9frFj0=OqFfea0dXdd9vqai=hGuQ8kuc9pgc9s8qqaq=dirpe0xb9q8qiLsFr0=vr0=vr0dc8meaabaqaciaacaGaaeqabaqabeGadaaakeaaiiGacuWF6oWAgaacaaaa@2E74@_1_(*ν *- 1)*q*^*ν *- 2^(1 - *q*)^2^.     (3)

In Eq. (3), the second term of RHS represents the flux by a mutation which simultaneously repairs the site carrying deleterious bit and destroys another site carrying a correct bit.

Eq. (3) is a necessary (but not sufficient) condition for the survival of G
 MathType@MTEF@5@5@+=feaafiart1ev1aaatCvAUfKttLearuWrP9MDH5MBPbIqV92AaeXatLxBI9gBaebbnrfifHhDYfgasaacH8akY=wiFfYdH8Gipec8Eeeu0xXdbba9frFj0=OqFfea0dXdd9vqai=hGuQ8kuc9pgc9s8qqaq=dirpe0xb9q8qiLsFr0=vr0=vr0dc8meaabaqaciaacaGaaeqabaqabeGadaaakeaat0uy0HwzTfgDPnwy1egaryqtHrhAL1wy0L2yHvdaiqaacqWFge=raaa@382C@_0 _without back mutations. However, since our objective is to examine whether the lethality of mutants nullifies the existence of an error-threshold, the necessary condition suffices. From Eq. (3), one obtains

q>κ˜1(ν−1)−κ˜1(ν−1)(f0/f1−1)1−f0/f1+κ˜1(ν−1)     (4)
 MathType@MTEF@5@5@+=feaafiart1ev1aaatCvAUfKttLearuWrP9MDH5MBPbIqV92AaeXatLxBI9gBaebbnrfifHhDYfgasaacH8akY=wiFfYdH8Gipec8Eeeu0xXdbba9frFj0=OqFfea0dXdd9vqai=hGuQ8kuc9pgc9s8qqaq=dirpe0xb9q8qiLsFr0=vr0=vr0dc8meaabaqaciaacaGaaeqabaqabeGadaaakeaacqWGXbqCcqGH+aGpdaWcaaqaaGGaciqb=P7aRzaaiaWaaSbaaSqaaiabigdaXaqabaGcdaqadaqaaiab=17aUjabgkHiTiabigdaXaGaayjkaiaawMcaaiabgkHiTmaakaaabaGaf8NUdSMbaGaadaWgaaWcbaGaeGymaedabeaakmaabmaabaGae8xVd4MaeyOeI0IaeGymaedacaGLOaGaayzkaaWaaeWaaeaacqWGMbGzdaWgaaWcbaGaeGimaadabeaakiabc+caViabdAgaMnaaBaaaleaacqaIXaqmaeqaaOGaeyOeI0IaeGymaedacaGLOaGaayzkaaaaleqaaaGcbaGaeGymaeJaeyOeI0IaemOzay2aaSbaaSqaaiabicdaWaqabaGccqGGVaWlcqWGMbGzdaWgaaWcbaGaeGymaedabeaakiabgUcaRiqb=P7aRzaaiaWaaSbaaSqaaiabigdaXaqabaGcdaqadaqaaiab=17aUjabgkHiTiabigdaXaGaayjkaiaawMcaaaaacaWLjaGaaCzcamaabmaabaGaeGinaqdacaGLOaGaayzkaaaaaa@5DDA@

as the condition for the survival of G
 MathType@MTEF@5@5@+=feaafiart1ev1aaatCvAUfKttLearuWrP9MDH5MBPbIqV92AaeXatLxBI9gBaebbnrfifHhDYfgasaacH8akY=wiFfYdH8Gipec8Eeeu0xXdbba9frFj0=OqFfea0dXdd9vqai=hGuQ8kuc9pgc9s8qqaq=dirpe0xb9q8qiLsFr0=vr0=vr0dc8meaabaqaciaacaGaaeqabaqabeGadaaakeaat0uy0HwzTfgDPnwy1egaryqtHrhAL1wy0L2yHvdaiqaacqWFge=raaa@382C@_0_. This sets the error-threshold (1 - *q*_min_).

The error-threshold derived as Eq. (4) disappears for *q*_min _< 1/2, where completely random replication suffices for the survival of G
 MathType@MTEF@5@5@+=feaafiart1ev1aaatCvAUfKttLearuWrP9MDH5MBPbIqV92AaeXatLxBI9gBaebbnrfifHhDYfgasaacH8akY=wiFfYdH8Gipec8Eeeu0xXdbba9frFj0=OqFfea0dXdd9vqai=hGuQ8kuc9pgc9s8qqaq=dirpe0xb9q8qiLsFr0=vr0=vr0dc8meaabaqaciaacaGaaeqabaqabeGadaaakeaat0uy0HwzTfgDPnwy1egaryqtHrhAL1wy0L2yHvdaiqaacqWFge=raaa@382C@_0_. The condition on lethality for *q*_min _< 1/2 is calculated as κ˜
 MathType@MTEF@5@5@+=feaafiart1ev1aaatCvAUfKttLearuWrP9MDH5MBPbIqV92AaeXatLxBI9gBaebbnrfifHhDYfgasaacH8akY=wiFfYdH8Gipec8Eeeu0xXdbba9frFj0=OqFfea0dXdd9vqai=hGuQ8kuc9pgc9s8qqaq=dirpe0xb9q8qiLsFr0=vr0=vr0dc8meaabaqaciaacaGaaeqabaqabeGadaaakeaaiiGacuWF6oWAgaacaaaa@2E74@_1 _< (*f*_0_/*f*_1 _- 1)/(*ν *- 1), and thus *κ *< (*f*_0_/*f*_1_)/*ν*[note that when *κ *approaches to (*f*_0_/*f*_1_)/*ν*, the enumerator and denominator of the RHS of Eq. (4) both approach to zero, and the RHS approaches to 1/2]. Due to the division by *ν*, *κ *must be quite small; i.e., the lethality (1 - *κ*) must be very high for this condition to hold. Furthermore, when *κ *= (*f*_0_/*f*_1_)/*ν*, the number of non-lethal genotypes in G
 MathType@MTEF@5@5@+=feaafiart1ev1aaatCvAUfKttLearuWrP9MDH5MBPbIqV92AaeXatLxBI9gBaebbnrfifHhDYfgasaacH8akY=wiFfYdH8Gipec8Eeeu0xXdbba9frFj0=OqFfea0dXdd9vqai=hGuQ8kuc9pgc9s8qqaq=dirpe0xb9q8qiLsFr0=vr0=vr0dc8meaabaqaciaacaGaaeqabaqabeGadaaakeaat0uy0HwzTfgDPnwy1egaryqtHrhAL1wy0L2yHvdaiqaacqWFge=raaa@382C@_1 _is *κν *= (*f*_0_/*f*_1_). Since *f*_0_/*f*_1 _is most likely close to 1 – in the population genetics terminology, *s *= ln(*f*_0_/*f*_1_) where *s *is the selection coefficient – this means that there is actually only one non-lethal genotype in G
 MathType@MTEF@5@5@+=feaafiart1ev1aaatCvAUfKttLearuWrP9MDH5MBPbIqV92AaeXatLxBI9gBaebbnrfifHhDYfgasaacH8akY=wiFfYdH8Gipec8Eeeu0xXdbba9frFj0=OqFfea0dXdd9vqai=hGuQ8kuc9pgc9s8qqaq=dirpe0xb9q8qiLsFr0=vr0=vr0dc8meaabaqaciaacaGaaeqabaqabeGadaaakeaat0uy0HwzTfgDPnwy1egaryqtHrhAL1wy0L2yHvdaiqaacqWFge=raaa@382C@_1_, which is a special case (in this case, the second term of the RHS of Eq. (3) disappears). Thus, we conclude that the error-threshold as defined above exists also with high lethality of mutants.

The essential reason of the existence of an error-threshold obviously comes from the term κ˜
 MathType@MTEF@5@5@+=feaafiart1ev1aaatCvAUfKttLearuWrP9MDH5MBPbIqV92AaeXatLxBI9gBaebbnrfifHhDYfgasaacH8akY=wiFfYdH8Gipec8Eeeu0xXdbba9frFj0=OqFfea0dXdd9vqai=hGuQ8kuc9pgc9s8qqaq=dirpe0xb9q8qiLsFr0=vr0=vr0dc8meaabaqaciaacaGaaeqabaqabeGadaaakeaaiiGacuWF6oWAgaacaaaa@2E74@_*d*_(*m*_*dd *_- *q*^*ν*^) in Eq. (2), i.e., the mutation within the same genotype class. Due to this term, the mutation rate of mutants is effectively lower than that of G
 MathType@MTEF@5@5@+=feaafiart1ev1aaatCvAUfKttLearuWrP9MDH5MBPbIqV92AaeXatLxBI9gBaebbnrfifHhDYfgasaacH8akY=wiFfYdH8Gipec8Eeeu0xXdbba9frFj0=OqFfea0dXdd9vqai=hGuQ8kuc9pgc9s8qqaq=dirpe0xb9q8qiLsFr0=vr0=vr0dc8meaabaqaciaacaGaaeqabaqabeGadaaakeaat0uy0HwzTfgDPnwy1egaryqtHrhAL1wy0L2yHvdaiqaacqWFge=raaa@382C@_0_. The mutation within the same genotype class is possible because more than one substitutions can happen per replication. As is clear from this analysis, multiple substitutions can play a significant role close to the error-threshold (see also [13]).

**Figure 1:**
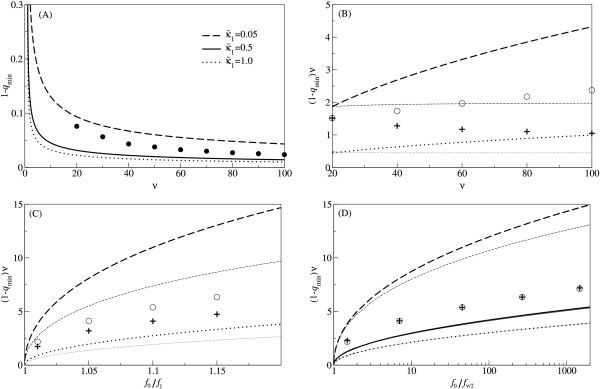
**Relationship between the error-threshold and various parameters**. Lines are calculated from Eq. (4) of the model without back mutation. The notation and parameters are as follows (unless stated otherwise): The broken line is for κ˜
 MathType@MTEF@5@5@+=feaafiart1ev1aaatCvAUfKttLearuWrP9MDH5MBPbIqV92AaeXatLxBI9gBaebbnrfifHhDYfgasaacH8akY=wiFfYdH8Gipec8Eeeu0xXdbba9frFj0=OqFfea0dXdd9vqai=hGuQ8kuc9pgc9s8qqaq=dirpe0xb9q8qiLsFr0=vr0=vr0dc8meaabaqaciaacaGaaeqabaqabeGadaaakeaaiiGacuWF6oWAgaacaaaa@2E74@_1 _= 0.05; the solid line is for κ˜
 MathType@MTEF@5@5@+=feaafiart1ev1aaatCvAUfKttLearuWrP9MDH5MBPbIqV92AaeXatLxBI9gBaebbnrfifHhDYfgasaacH8akY=wiFfYdH8Gipec8Eeeu0xXdbba9frFj0=OqFfea0dXdd9vqai=hGuQ8kuc9pgc9s8qqaq=dirpe0xb9q8qiLsFr0=vr0=vr0dc8meaabaqaciaacaGaaeqabaqabeGadaaakeaaiiGacuWF6oWAgaacaaaa@2E74@_1 _= 0.5; the dotted line is for κ˜
 MathType@MTEF@5@5@+=feaafiart1ev1aaatCvAUfKttLearuWrP9MDH5MBPbIqV92AaeXatLxBI9gBaebbnrfifHhDYfgasaacH8akY=wiFfYdH8Gipec8Eeeu0xXdbba9frFj0=OqFfea0dXdd9vqai=hGuQ8kuc9pgc9s8qqaq=dirpe0xb9q8qiLsFr0=vr0=vr0dc8meaabaqaciaacaGaaeqabaqabeGadaaakeaaiiGacuWF6oWAgaacaaaa@2E74@_1 _= 1.0; *f*_0_/*f*_1 _= 0.99^-1^. Points (circles and plus signs) are calculated through the comparison of the first and second greatest eigen values of the model with back mutation [see "How high must lethality be to introduce an error-threshold" and Fig. 4(A) for details]. *κ *= 0.05 (the same as in Fig. 3) and *f*_*d *_= 0.99^*d*^, unless stated otherwise. Note that Eq. (4) is the necessary condition of the error-threshold without back mutation, and thus, it overestimates 1 - *q*_min _compared to the calculation through the comparison between the eigen values. **(A) **Relationship between the error-threshold and sequence length. The graph shows that the error-threshold becomes smaller as *ν *increases. Note the faster decrease shown by the circles compared to that by the dashed line. Although there is a scaling between *ν *and κ˜
 MathType@MTEF@5@5@+=feaafiart1ev1aaatCvAUfKttLearuWrP9MDH5MBPbIqV92AaeXatLxBI9gBaebbnrfifHhDYfgasaacH8akY=wiFfYdH8Gipec8Eeeu0xXdbba9frFj0=OqFfea0dXdd9vqai=hGuQ8kuc9pgc9s8qqaq=dirpe0xb9q8qiLsFr0=vr0=vr0dc8meaabaqaciaacaGaaeqabaqabeGadaaakeaaiiGacuWF6oWAgaacaaaa@2E74@ for the lines, as seen from κ˜
 MathType@MTEF@5@5@+=feaafiart1ev1aaatCvAUfKttLearuWrP9MDH5MBPbIqV92AaeXatLxBI9gBaebbnrfifHhDYfgasaacH8akY=wiFfYdH8Gipec8Eeeu0xXdbba9frFj0=OqFfea0dXdd9vqai=hGuQ8kuc9pgc9s8qqaq=dirpe0xb9q8qiLsFr0=vr0=vr0dc8meaabaqaciaacaGaaeqabaqabeGadaaakeaaiiGacuWF6oWAgaacaaaa@2E74@(*ν *- 1) term in Eq. (4), this is merely due to the fact that only G
 MathType@MTEF@5@5@+=feaafiart1ev1aaatCvAUfKttLearuWrP9MDH5MBPbIqV92AaeXatLxBI9gBaebbnrfifHhDYfgasaacH8akY=wiFfYdH8Gipec8Eeeu0xXdbba9frFj0=OqFfea0dXdd9vqai=hGuQ8kuc9pgc9s8qqaq=dirpe0xb9q8qiLsFr0=vr0=vr0dc8meaabaqaciaacaGaaeqabaqabeGadaaakeaat0uy0HwzTfgDPnwy1egaryqtHrhAL1wy0L2yHvdaiqaacqWFge=raaa@382C@_0 _and G
 MathType@MTEF@5@5@+=feaafiart1ev1aaatCvAUfKttLearuWrP9MDH5MBPbIqV92AaeXatLxBI9gBaebbnrfifHhDYfgasaacH8akY=wiFfYdH8Gipec8Eeeu0xXdbba9frFj0=OqFfea0dXdd9vqai=hGuQ8kuc9pgc9s8qqaq=dirpe0xb9q8qiLsFr0=vr0=vr0dc8meaabaqaciaacaGaaeqabaqabeGadaaakeaat0uy0HwzTfgDPnwy1egaryqtHrhAL1wy0L2yHvdaiqaacqWFge=raaa@382C@_1 _are considered in Eq. (4). **(B) **Relationship between the maximum tolerable genomic mutation rate per replication [i.e., (1 - *q*_min_)*ν*] and *ν*. The graph shows that (1 - *q*_min_)*ν *increases as *ν *increases if *f*_0_/*f*_1 _is kept constant (the thick lines and the circles), but also shows that this does not hold if *f*_0_/fν2
 MathType@MTEF@5@5@+=feaafiart1ev1aaatCvAUfKttLearuWrP9MDH5MBPbIqV92AaeXatLxBI9gBaebbnrfifHhDYfgasaacH8akY=wiFfYdH8Gipec8Eeeu0xXdbba9frFj0=OqFfea0dXdd9vqai=hGuQ8kuc9pgc9s8qqaq=dirpe0xb9q8qiLsFr0=vr0=vr0dc8meaabaqaciaacaGaaeqabaqabeGadaaakeaacqWGMbGzdaWgaaWcbaWaaSaaaeaaiiGacqWF9oGBaeaacqaIYaGmaaaabeaaaaa@30EE@ is kept constant (the thin lines and the plus signs). *f*_0_/fν2
 MathType@MTEF@5@5@+=feaafiart1ev1aaatCvAUfKttLearuWrP9MDH5MBPbIqV92AaeXatLxBI9gBaebbnrfifHhDYfgasaacH8akY=wiFfYdH8Gipec8Eeeu0xXdbba9frFj0=OqFfea0dXdd9vqai=hGuQ8kuc9pgc9s8qqaq=dirpe0xb9q8qiLsFr0=vr0=vr0dc8meaabaqaciaacaGaaeqabaqabeGadaaakeaacqWGMbGzdaWgaaWcbaWaaSaaaeaaiiGacqWF9oGBaeaacqaIYaGmaaaabeaaaaa@30EE@ represents the relative reproductive advantage of the fittest genotype compared to the perfectly randomized genotype which is not lethal. The thin lines are obtained by setting *f*_0_/fν2
 MathType@MTEF@5@5@+=feaafiart1ev1aaatCvAUfKttLearuWrP9MDH5MBPbIqV92AaeXatLxBI9gBaebbnrfifHhDYfgasaacH8akY=wiFfYdH8Gipec8Eeeu0xXdbba9frFj0=OqFfea0dXdd9vqai=hGuQ8kuc9pgc9s8qqaq=dirpe0xb9q8qiLsFr0=vr0=vr0dc8meaabaqaciaacaGaaeqabaqabeGadaaakeaacqWGMbGzdaWgaaWcbaWaaSaaaeaaiiGacqWF9oGBaeaacqaIYaGmaaaabeaaaaa@30EE@ to a constant value such that it coincides with the corresponding thick line at *ν *= 20; i.e., f0/fν2=0.99−202
 MathType@MTEF@5@5@+=feaafiart1ev1aaatCvAUfKttLearuWrP9MDH5MBPbIqV92AaeXatLxBI9gBaebbnrfifHhDYfgasaacH8akY=wiFfYdH8Gipec8Eeeu0xXdbba9frFj0=OqFfea0dXdd9vqai=hGuQ8kuc9pgc9s8qqaq=dirpe0xb9q8qiLsFr0=vr0=vr0dc8meaabaqaciaacaGaaeqabaqabeGadaaakeaacqWGMbGzdaWgaaWcbaGaeGimaadabeaakiabc+caViabdAgaMnaaBaaaleaadaWcaaqaaGGaciab=17aUbqaaiabikdaYaaaaeqaaOGaeyypa0JaeGimaaJaeiOla4IaeGyoaKJaeGyoaKZaaWbaaSqabeaacqGHsisldaWcaaqaaiabikdaYiabicdaWaqaaiabikdaYaaaaaaaaa@3D2B@, and *f*_0_/*f*_1 _is calculated as 0.99−20ν
 MathType@MTEF@5@5@+=feaafiart1ev1aaatCvAUfKttLearuWrP9MDH5MBPbIqV92AaeXatLxBI9gBaebbnrfifHhDYfgasaacH8akY=wiFfYdH8Gipec8Eeeu0xXdbba9frFj0=OqFfea0dXdd9vqai=hGuQ8kuc9pgc9s8qqaq=dirpe0xb9q8qiLsFr0=vr0=vr0dc8meaabaqaciaacaGaaeqabaqabeGadaaakeaacqaIWaamcqGGUaGlcqaI5aqocqaI5aqodaahaaWcbeqaaiabgkHiTmaalaaabaGaeGOmaiJaeGimaadabaacciGae8xVd4gaaaaaaaa@3547@ by assuming a multiplicative fitness landscape. The circles are for a constant *f*_0_/*f*_1_. The plus signs are obtained by setting *f*_0_/fν2
 MathType@MTEF@5@5@+=feaafiart1ev1aaatCvAUfKttLearuWrP9MDH5MBPbIqV92AaeXatLxBI9gBaebbnrfifHhDYfgasaacH8akY=wiFfYdH8Gipec8Eeeu0xXdbba9frFj0=OqFfea0dXdd9vqai=hGuQ8kuc9pgc9s8qqaq=dirpe0xb9q8qiLsFr0=vr0=vr0dc8meaabaqaciaacaGaaeqabaqabeGadaaakeaacqWGMbGzdaWgaaWcbaWaaSaaaeaaiiGacqWF9oGBaeaacqaIYaGmaaaabeaaaaa@30EE@ to a constant value such that it coincides with the circles at *ν *= 20 [f0/fν2=0.99−202
 MathType@MTEF@5@5@+=feaafiart1ev1aaatCvAUfKttLearuWrP9MDH5MBPbIqV92AaeXatLxBI9gBaebbnrfifHhDYfgasaacH8akY=wiFfYdH8Gipec8Eeeu0xXdbba9frFj0=OqFfea0dXdd9vqai=hGuQ8kuc9pgc9s8qqaq=dirpe0xb9q8qiLsFr0=vr0=vr0dc8meaabaqaciaacaGaaeqabaqabeGadaaakeaacqWGMbGzdaWgaaWcbaGaeGimaadabeaakiabc+caViabdAgaMnaaBaaaleaadaWcaaqaaGGaciab=17aUbqaaiabikdaYaaaaeqaaOGaeyypa0JaeGimaaJaeiOla4IaeGyoaKJaeGyoaKZaaWbaaSqabeaacqGHsisldaWcaaqaaiabikdaYiabicdaWaqaaiabikdaYaaaaaaaaa@3D2B@; thus, *f*_*d *_= 0.99^(20/*ν*)*d*^]. (The lines for κ˜
 MathType@MTEF@5@5@+=feaafiart1ev1aaatCvAUfKttLearuWrP9MDH5MBPbIqV92AaeXatLxBI9gBaebbnrfifHhDYfgasaacH8akY=wiFfYdH8Gipec8Eeeu0xXdbba9frFj0=OqFfea0dXdd9vqai=hGuQ8kuc9pgc9s8qqaq=dirpe0xb9q8qiLsFr0=vr0=vr0dc8meaabaqaciaacaGaaeqabaqabeGadaaakeaaiiGacuWF6oWAgaacaaaa@2E74@_1 _= 0.5 are not shown for visibility.) **(C) **Relationship between (1 - *q*_min_)*ν *and the intensity of selection *f*_0_/*f*_1_. The graph shows that for a given value of *f*_0_/*f*_1_, a longer sequence can tolerate a greater (1 - *q*_min_)*ν*. In other words, for a given value of (1 - *q*)*ν*, a longer sequence can be maintained (i.e., 1 - *q *is below the error-threshold) for a smaller value of *f*_0_/*f*_1_. The thick lines and circles are for *ν *= 80. The thin lines and plus signs are for *ν *= 40. For all plots, *f*_*d *_= (*f*_0_/*f*_1_)^*-d*^. (The lines for κ˜
 MathType@MTEF@5@5@+=feaafiart1ev1aaatCvAUfKttLearuWrP9MDH5MBPbIqV92AaeXatLxBI9gBaebbnrfifHhDYfgasaacH8akY=wiFfYdH8Gipec8Eeeu0xXdbba9frFj0=OqFfea0dXdd9vqai=hGuQ8kuc9pgc9s8qqaq=dirpe0xb9q8qiLsFr0=vr0=vr0dc8meaabaqaciaacaGaaeqabaqabeGadaaakeaaiiGacuWF6oWAgaacaaaa@2E74@_1 _= 0.5 are not shown for visibility) **(D) **Relationship between (1 - *q*_min_)*ν *and *f*_0_/fν2
 MathType@MTEF@5@5@+=feaafiart1ev1aaatCvAUfKttLearuWrP9MDH5MBPbIqV92AaeXatLxBI9gBaebbnrfifHhDYfgasaacH8akY=wiFfYdH8Gipec8Eeeu0xXdbba9frFj0=OqFfea0dXdd9vqai=hGuQ8kuc9pgc9s8qqaq=dirpe0xb9q8qiLsFr0=vr0=vr0dc8meaabaqaciaacaGaaeqabaqabeGadaaakeaacqWGMbGzdaWgaaWcbaWaaSaaaeaaiiGacqWF9oGBaeaacqaIYaGmaaaabeaaaaa@30EE@. The graph shows that for a given value of *f*_0_/fν2
 MathType@MTEF@5@5@+=feaafiart1ev1aaatCvAUfKttLearuWrP9MDH5MBPbIqV92AaeXatLxBI9gBaebbnrfifHhDYfgasaacH8akY=wiFfYdH8Gipec8Eeeu0xXdbba9frFj0=OqFfea0dXdd9vqai=hGuQ8kuc9pgc9s8qqaq=dirpe0xb9q8qiLsFr0=vr0=vr0dc8meaabaqaciaacaGaaeqabaqabeGadaaakeaacqWGMbGzdaWgaaWcbaWaaSaaaeaaiiGacqWF9oGBaeaacqaIYaGmaaaabeaaaaa@30EE@, (1 - *q*_min_)*ν *does not differ between different sequence length (this is comparable to the results of [3, 14], and note a parallelism between *f*_0_/fν2
 MathType@MTEF@5@5@+=feaafiart1ev1aaatCvAUfKttLearuWrP9MDH5MBPbIqV92AaeXatLxBI9gBaebbnrfifHhDYfgasaacH8akY=wiFfYdH8Gipec8Eeeu0xXdbba9frFj0=OqFfea0dXdd9vqai=hGuQ8kuc9pgc9s8qqaq=dirpe0xb9q8qiLsFr0=vr0=vr0dc8meaabaqaciaacaGaaeqabaqabeGadaaakeaacqWGMbGzdaWgaaWcbaWaaSaaaeaaiiGacqWF9oGBaeaacqaIYaGmaaaabeaaaaa@30EE@ and the superiority parameter *σ *defined there). For *κ *= 0.05 (dashed lines), there is still a noticeable difference, but this is due to the fact that only G
 MathType@MTEF@5@5@+=feaafiart1ev1aaatCvAUfKttLearuWrP9MDH5MBPbIqV92AaeXatLxBI9gBaebbnrfifHhDYfgasaacH8akY=wiFfYdH8Gipec8Eeeu0xXdbba9frFj0=OqFfea0dXdd9vqai=hGuQ8kuc9pgc9s8qqaq=dirpe0xb9q8qiLsFr0=vr0=vr0dc8meaabaqaciaacaGaaeqabaqabeGadaaakeaat0uy0HwzTfgDPnwy1egaryqtHrhAL1wy0L2yHvdaiqaacqWFge=raaa@382C@_0 _and G
 MathType@MTEF@5@5@+=feaafiart1ev1aaatCvAUfKttLearuWrP9MDH5MBPbIqV92AaeXatLxBI9gBaebbnrfifHhDYfgasaacH8akY=wiFfYdH8Gipec8Eeeu0xXdbba9frFj0=OqFfea0dXdd9vqai=hGuQ8kuc9pgc9s8qqaq=dirpe0xb9q8qiLsFr0=vr0=vr0dc8meaabaqaciaacaGaaeqabaqabeGadaaakeaat0uy0HwzTfgDPnwy1egaryqtHrhAL1wy0L2yHvdaiqaacqWFge=raaa@382C@_1 _are considered to obtain them although (1 - *q*_min_)*ν *is large. [compare this result with the circles and plus signs, which are obtained by considering all G
 MathType@MTEF@5@5@+=feaafiart1ev1aaatCvAUfKttLearuWrP9MDH5MBPbIqV92AaeXatLxBI9gBaebbnrfifHhDYfgasaacH8akY=wiFfYdH8Gipec8Eeeu0xXdbba9frFj0=OqFfea0dXdd9vqai=hGuQ8kuc9pgc9s8qqaq=dirpe0xb9q8qiLsFr0=vr0=vr0dc8meaabaqaciaacaGaaeqabaqabeGadaaakeaat0uy0HwzTfgDPnwy1egaryqtHrhAL1wy0L2yHvdaiqaacqWFge=raaa@382C@_*d *_(0 ≤ *d *≤ *ν*) for almost the same value of *κ*]. For all plots, *f*_*d *_= (*f*_0_/fν2
 MathType@MTEF@5@5@+=feaafiart1ev1aaatCvAUfKttLearuWrP9MDH5MBPbIqV92AaeXatLxBI9gBaebbnrfifHhDYfgasaacH8akY=wiFfYdH8Gipec8Eeeu0xXdbba9frFj0=OqFfea0dXdd9vqai=hGuQ8kuc9pgc9s8qqaq=dirpe0xb9q8qiLsFr0=vr0=vr0dc8meaabaqaciaacaGaaeqabaqabeGadaaakeaacqWGMbGzdaWgaaWcbaWaaSaaaeaaiiGacqWF9oGBaeaacqaIYaGmaaaabeaaaaa@30EE@)^(-2/*ν*)*d*^. The notation and the rest of parameters are the same as those in (C). (the lines for *ν *= 40 and *ν *= 80 are almost on top of each other for κ˜
 MathType@MTEF@5@5@+=feaafiart1ev1aaatCvAUfKttLearuWrP9MDH5MBPbIqV92AaeXatLxBI9gBaebbnrfifHhDYfgasaacH8akY=wiFfYdH8Gipec8Eeeu0xXdbba9frFj0=OqFfea0dXdd9vqai=hGuQ8kuc9pgc9s8qqaq=dirpe0xb9q8qiLsFr0=vr0=vr0dc8meaabaqaciaacaGaaeqabaqabeGadaaakeaaiiGacuWF6oWAgaacaaaa@2E74@_1 _= 0.5 and 1.0.)

A significant aspect of error-threshold is that it hampers the accumulation of information through increasing the genome size of the replicator. This is the case if it becomes harder to maintain G
 MathType@MTEF@5@5@+=feaafiart1ev1aaatCvAUfKttLearuWrP9MDH5MBPbIqV92AaeXatLxBI9gBaebbnrfifHhDYfgasaacH8akY=wiFfYdH8Gipec8Eeeu0xXdbba9frFj0=OqFfea0dXdd9vqai=hGuQ8kuc9pgc9s8qqaq=dirpe0xb9q8qiLsFr0=vr0=vr0dc8meaabaqaciaacaGaaeqabaqabeGadaaakeaat0uy0HwzTfgDPnwy1egaryqtHrhAL1wy0L2yHvdaiqaacqWFge=raaa@382C@_0_, a genome with all sites carrying a correct character, by Darwinian selection as the genome size increases. In the next, we examine the effect of lethality on the relationship between the error-threshold and replicator genome size (*ν*). In Fig. 1(A), the error-threshold derived as Eq. (4) is plotted against sequence length *ν *for various values of κ˜
 MathType@MTEF@5@5@+=feaafiart1ev1aaatCvAUfKttLearuWrP9MDH5MBPbIqV92AaeXatLxBI9gBaebbnrfifHhDYfgasaacH8akY=wiFfYdH8Gipec8Eeeu0xXdbba9frFj0=OqFfea0dXdd9vqai=hGuQ8kuc9pgc9s8qqaq=dirpe0xb9q8qiLsFr0=vr0=vr0dc8meaabaqaciaacaGaaeqabaqabeGadaaakeaaiiGacuWF6oWAgaacaaaa@2E74@ by assuming that *f*_0_/*f*_1 _is constant for different values of *ν*. As shown in Fig. 1(A), first, a decrease in κ˜
 MathType@MTEF@5@5@+=feaafiart1ev1aaatCvAUfKttLearuWrP9MDH5MBPbIqV92AaeXatLxBI9gBaebbnrfifHhDYfgasaacH8akY=wiFfYdH8Gipec8Eeeu0xXdbba9frFj0=OqFfea0dXdd9vqai=hGuQ8kuc9pgc9s8qqaq=dirpe0xb9q8qiLsFr0=vr0=vr0dc8meaabaqaciaacaGaaeqabaqabeGadaaakeaaiiGacuWF6oWAgaacaaaa@2E74@ increases the error-threshold. Second, however, the negative dependency of the error-threshold on *ν *persists for each value of κ˜
 MathType@MTEF@5@5@+=feaafiart1ev1aaatCvAUfKttLearuWrP9MDH5MBPbIqV92AaeXatLxBI9gBaebbnrfifHhDYfgasaacH8akY=wiFfYdH8Gipec8Eeeu0xXdbba9frFj0=OqFfea0dXdd9vqai=hGuQ8kuc9pgc9s8qqaq=dirpe0xb9q8qiLsFr0=vr0=vr0dc8meaabaqaciaacaGaaeqabaqabeGadaaakeaaiiGacuWF6oWAgaacaaaa@2E74@; i.e., the condition on *q *for G
 MathType@MTEF@5@5@+=feaafiart1ev1aaatCvAUfKttLearuWrP9MDH5MBPbIqV92AaeXatLxBI9gBaebbnrfifHhDYfgasaacH8akY=wiFfYdH8Gipec8Eeeu0xXdbba9frFj0=OqFfea0dXdd9vqai=hGuQ8kuc9pgc9s8qqaq=dirpe0xb9q8qiLsFr0=vr0=vr0dc8meaabaqaciaacaGaaeqabaqabeGadaaakeaat0uy0HwzTfgDPnwy1egaryqtHrhAL1wy0L2yHvdaiqaacqWFge=raaa@382C@_0_'s survival becomes harder as *ν *increases. The first result is understood because the effect of mutation between the same genotype class, κ˜
 MathType@MTEF@5@5@+=feaafiart1ev1aaatCvAUfKttLearuWrP9MDH5MBPbIqV92AaeXatLxBI9gBaebbnrfifHhDYfgasaacH8akY=wiFfYdH8Gipec8Eeeu0xXdbba9frFj0=OqFfea0dXdd9vqai=hGuQ8kuc9pgc9s8qqaq=dirpe0xb9q8qiLsFr0=vr0=vr0dc8meaabaqaciaacaGaaeqabaqabeGadaaakeaaiiGacuWF6oWAgaacaaaa@2E74@_1_(*m*_11 _- *q*^*ν*^), becomes smaller as κ˜
 MathType@MTEF@5@5@+=feaafiart1ev1aaatCvAUfKttLearuWrP9MDH5MBPbIqV92AaeXatLxBI9gBaebbnrfifHhDYfgasaacH8akY=wiFfYdH8Gipec8Eeeu0xXdbba9frFj0=OqFfea0dXdd9vqai=hGuQ8kuc9pgc9s8qqaq=dirpe0xb9q8qiLsFr0=vr0=vr0dc8meaabaqaciaacaGaaeqabaqabeGadaaakeaaiiGacuWF6oWAgaacaaaa@2E74@ decreases. The second result is explained as follows. As *ν *increases, the average number of substitutions per sequence per replication, *ν*(1 - *q*), increases. Thus, the contribution of mutation between the same genotype class to the net reproduction rate, *f*_1_κ˜
 MathType@MTEF@5@5@+=feaafiart1ev1aaatCvAUfKttLearuWrP9MDH5MBPbIqV92AaeXatLxBI9gBaebbnrfifHhDYfgasaacH8akY=wiFfYdH8Gipec8Eeeu0xXdbba9frFj0=OqFfea0dXdd9vqai=hGuQ8kuc9pgc9s8qqaq=dirpe0xb9q8qiLsFr0=vr0=vr0dc8meaabaqaciaacaGaaeqabaqabeGadaaakeaaiiGacuWF6oWAgaacaaaa@2E74@_1_(*m*_11 _- *q*^*ν*^), becomes relatively greater compared to that of perfect replication, *f*_1_*q*^*ν*^. This results in the greater advantage of mutants, and hence the error-threshold becomes more severe.

For the result in the previous paragraph to hold, it must be assumed that *f*_0_/*f*_1 _(intensity of selection) does not increase as *ν *increases. However, this is fulfilled rather easily: The constancy of *f*_0_/*f*_1_, in response to the increase of *ν*, assumes that every addition of one correct character to G
 MathType@MTEF@5@5@+=feaafiart1ev1aaatCvAUfKttLearuWrP9MDH5MBPbIqV92AaeXatLxBI9gBaebbnrfifHhDYfgasaacH8akY=wiFfYdH8Gipec8Eeeu0xXdbba9frFj0=OqFfea0dXdd9vqai=hGuQ8kuc9pgc9s8qqaq=dirpe0xb9q8qiLsFr0=vr0=vr0dc8meaabaqaciaacaGaaeqabaqabeGadaaakeaat0uy0HwzTfgDPnwy1egaryqtHrhAL1wy0L2yHvdaiqaacqWFge=raaa@382C@_0 _results in the multiplicative increase of G
 MathType@MTEF@5@5@+=feaafiart1ev1aaatCvAUfKttLearuWrP9MDH5MBPbIqV92AaeXatLxBI9gBaebbnrfifHhDYfgasaacH8akY=wiFfYdH8Gipec8Eeeu0xXdbba9frFj0=OqFfea0dXdd9vqai=hGuQ8kuc9pgc9s8qqaq=dirpe0xb9q8qiLsFr0=vr0=vr0dc8meaabaqaciaacaGaaeqabaqabeGadaaakeaat0uy0HwzTfgDPnwy1egaryqtHrhAL1wy0L2yHvdaiqaacqWFge=raaa@382C@_0_'s reproduction rate; however, this is hardly realistic, not to mention the increase of *f*_0_/*f*_1 _(cf. [4,12]). In the next paragraph, this point is further elaborated.

A consequence of constant *f*_0_/*f*_1 _relative to a change in *ν *can be seen in Fig. 1(B), which shows that a longer sequence can tolerate, actually, a greater genomic mutation rate per replication, in contrast to the results of [3,14]. This is illustrated from a different aspect by Fig. 1(C), as it shows that for a given genomic mutation rate per replication, a longer genome can be maintained with a smaller value of *f*_0_/*f*_1 _(see the explanation in the caption too). These observations are due to the fact that, under the assumption of constant *f*_0_/*f*_1_, the reproductive advantage of G
 MathType@MTEF@5@5@+=feaafiart1ev1aaatCvAUfKttLearuWrP9MDH5MBPbIqV92AaeXatLxBI9gBaebbnrfifHhDYfgasaacH8akY=wiFfYdH8Gipec8Eeeu0xXdbba9frFj0=OqFfea0dXdd9vqai=hGuQ8kuc9pgc9s8qqaq=dirpe0xb9q8qiLsFr0=vr0=vr0dc8meaabaqaciaacaGaaeqabaqabeGadaaakeaat0uy0HwzTfgDPnwy1egaryqtHrhAL1wy0L2yHvdaiqaacqWFge=raaa@382C@_0 _relative to the completely randomized non-lethal genotypes Gν2
 MathType@MTEF@5@5@+=feaafiart1ev1aaatCvAUfKttLearuWrP9MDH5MBPbIqV92AaeXatLxBI9gBaebbnrfifHhDYfgasaacH8akY=wiFfYdH8Gipec8Eeeu0xXdbba9frFj0=OqFfea0dXdd9vqai=hGuQ8kuc9pgc9s8qqaq=dirpe0xb9q8qiLsFr0=vr0=vr0dc8meaabaqaciaacaGaaeqabaqabeGadaaakeaat0uy0HwzTfgDPnwy1egaryqtHrhAL1wy0L2yHvdaiqaacqWFge=rdaWgaaWcbaWaaSaaaeaaiiGacqGF9oGBaeaacqaIYaGmaaaabeaaaaa@3B18@ (in other words, relative to the consensus sequence when 1 - *q *= 0.5) increases exponentially as *ν *increases. If this relative advantage of G
 MathType@MTEF@5@5@+=feaafiart1ev1aaatCvAUfKttLearuWrP9MDH5MBPbIqV92AaeXatLxBI9gBaebbnrfifHhDYfgasaacH8akY=wiFfYdH8Gipec8Eeeu0xXdbba9frFj0=OqFfea0dXdd9vqai=hGuQ8kuc9pgc9s8qqaq=dirpe0xb9q8qiLsFr0=vr0=vr0dc8meaabaqaciaacaGaaeqabaqabeGadaaakeaat0uy0HwzTfgDPnwy1egaryqtHrhAL1wy0L2yHvdaiqaacqWFge=raaa@382C@_0 _(i.e., *f*_0_/fν2
 MathType@MTEF@5@5@+=feaafiart1ev1aaatCvAUfKttLearuWrP9MDH5MBPbIqV92AaeXatLxBI9gBaebbnrfifHhDYfgasaacH8akY=wiFfYdH8Gipec8Eeeu0xXdbba9frFj0=OqFfea0dXdd9vqai=hGuQ8kuc9pgc9s8qqaq=dirpe0xb9q8qiLsFr0=vr0=vr0dc8meaabaqaciaacaGaaeqabaqabeGadaaakeaacqWGMbGzdaWgaaWcbaWaaSaaaeaaiiGacqWF9oGBaeaacqaIYaGmaaaabeaaaaa@30EE@) is kept constant, the results qualitatively differ as shown in Fig. 1(B,D). In particular, the maximum tolerable genomic mutation rate per replication [i.e., (1 - *q*_min_)*ν*] becomes independent of *ν*, as is the case in [3,14]. Back to the results of the second-to-last paragraph, the fact that the negative dependency of the error-threshold on *ν *holds even in the case of constant *f*_0_/*f*_1_, while (1 - *q*_min_)*ν*, however, increases as *ν *increases, emphasizes the generality of this result.

### Error-threshold in the model with back mutations

In this section, we will examine the effect of lethality on the distribution of the population in the genotype space with the full consideration of back mutations. It is assumed in the following that the fitness landscape is multiplicative; i.e., *f*_*d *_decreases exponentially with *d *(*f*_*d *_= 0.99^*d*^).

**Figure 2:**
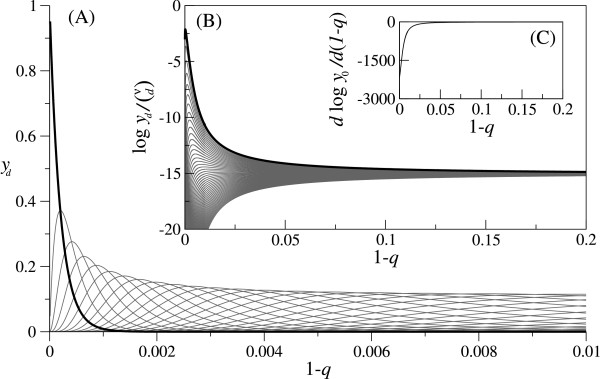
**Equilibrium population distribution without lethality**. The stationary solution of Eq. (2) without lethality. The figure shows the delocalization of the population from the fittest genotype for high error rates and the absence of the error-threshold. **(A) **The population fraction of each genotype class (*y*_*d*_) is plotted against the error rate (1 - *q*). The black line is for *y*_0_. The gray lines are for *y*_*d *_(0 <*d *≤ *ν*). A succession of the genotype class is observed as 1 - *q *increase (G
 MathType@MTEF@5@5@+=feaafiart1ev1aaatCvAUfKttLearuWrP9MDH5MBPbIqV92AaeXatLxBI9gBaebbnrfifHhDYfgasaacH8akY=wiFfYdH8Gipec8Eeeu0xXdbba9frFj0=OqFfea0dXdd9vqai=hGuQ8kuc9pgc9s8qqaq=dirpe0xb9q8qiLsFr0=vr0=vr0dc8meaabaqaciaacaGaaeqabaqabeGadaaakeaat0uy0HwzTfgDPnwy1egaryqtHrhAL1wy0L2yHvdaiqaacqWFge=raaa@382C@_0 _is maximum at 1 - *q *≈ 0, then G
 MathType@MTEF@5@5@+=feaafiart1ev1aaatCvAUfKttLearuWrP9MDH5MBPbIqV92AaeXatLxBI9gBaebbnrfifHhDYfgasaacH8akY=wiFfYdH8Gipec8Eeeu0xXdbba9frFj0=OqFfea0dXdd9vqai=hGuQ8kuc9pgc9s8qqaq=dirpe0xb9q8qiLsFr0=vr0=vr0dc8meaabaqaciaacaGaaeqabaqabeGadaaakeaat0uy0HwzTfgDPnwy1egaryqtHrhAL1wy0L2yHvdaiqaacqWFge=raaa@382C@_1_, G
 MathType@MTEF@5@5@+=feaafiart1ev1aaatCvAUfKttLearuWrP9MDH5MBPbIqV92AaeXatLxBI9gBaebbnrfifHhDYfgasaacH8akY=wiFfYdH8Gipec8Eeeu0xXdbba9frFj0=OqFfea0dXdd9vqai=hGuQ8kuc9pgc9s8qqaq=dirpe0xb9q8qiLsFr0=vr0=vr0dc8meaabaqaciaacaGaaeqabaqabeGadaaakeaat0uy0HwzTfgDPnwy1egaryqtHrhAL1wy0L2yHvdaiqaacqWFge=raaa@382C@_2_, G
 MathType@MTEF@5@5@+=feaafiart1ev1aaatCvAUfKttLearuWrP9MDH5MBPbIqV92AaeXatLxBI9gBaebbnrfifHhDYfgasaacH8akY=wiFfYdH8Gipec8Eeeu0xXdbba9frFj0=OqFfea0dXdd9vqai=hGuQ8kuc9pgc9s8qqaq=dirpe0xb9q8qiLsFr0=vr0=vr0dc8meaabaqaciaacaGaaeqabaqabeGadaaakeaat0uy0HwzTfgDPnwy1egaryqtHrhAL1wy0L2yHvdaiqaacqWFge=raaa@382C@_3_, ⋯ as 1 - *q *increases.) **(B) **The logarithm of the population fraction of each genotype, log(*y*_*d*_/(κ(νd)
 MathType@MTEF@5@5@+=feaafiart1ev1aaatCvAUfKttLearuWrP9MDH5MBPbIqV92AaeXatLxBI9gBaebbnrfifHhDYfgasaacH8akY=wiFfYdH8Gipec8Eeeu0xXdbba9frFj0=OqFfea0dXdd9vqai=hGuQ8kuc9pgc9s8qqaq=dirpe0xb9q8qiLsFr0=vr0=vr0dc8meaabaqaciaacaGaaeqabaqabeGadaaakeaaiiGacqWF6oWAdaqadaqaauaabeqaceaaaeaacqWF9oGBaeaacqWGKbazaaaacaGLOaGaayzkaaaaaa@32FF@)), is plotted against 1 - *q *(instead of that of a genotype class). The black line is for *d *= 0. The gray lines are for 0 <*d *≤ *ν *[from top to bottom, lines are for *d *= 0,1, 2, ...]. The graph depicts the convergence of the population fraction of every genotype to the limit (∑d=0ν(νd))−1
 MathType@MTEF@5@5@+=feaafiart1ev1aaatCvAUfKttLearuWrP9MDH5MBPbIqV92AaeXatLxBI9gBaebbnrfifHhDYfgasaacH8akY=wiFfYdH8Gipec8Eeeu0xXdbba9frFj0=OqFfea0dXdd9vqai=hGuQ8kuc9pgc9s8qqaq=dirpe0xb9q8qiLsFr0=vr0=vr0dc8meaabaqaciaacaGaaeqabaqabeGadaaakeaadaqadaqaamaaqadabaWaaeWaaeaafaqabeGabaaabaacciGae8xVd4gabaGaemizaqgaaaGaayjkaiaawMcaaaWcbaGaemizaqMaeyypa0JaeGimaadabaGae8xVd4ganiabggHiLdaakiaawIcacaGLPaaadaahaaWcbeqaaiabgkHiTiabigdaXaaaaaa@3BE9@, which is the population fraction of a genotype for *q *= 0.5. **(C) ***d*log *y*_0_/*d*(1 - *q*) is plotted against 1 - *q*. For all graphs, the parameters are as follows, *ν *= 50. *f*_*d *_= 0.99^*d*^. *κ *= 1.

**Figure 3:**
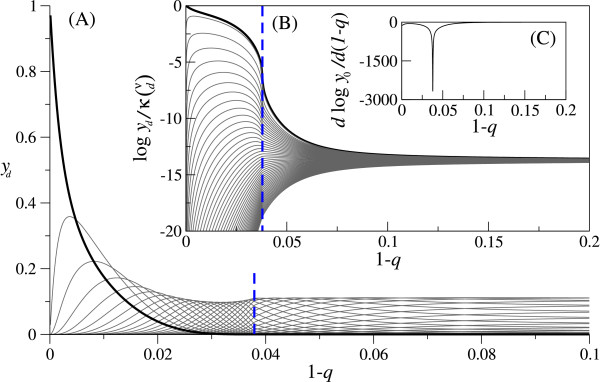
**Equilibrium population distribution with high lethality**. Similar plots as in Fig. 2, but with high lethality of mutants. The figure displays the existence of the error-threshold, and the delocalization of the population from the fittest genotype. The blue dashed line is placed in Fig. 3(A,B) at the error rate for which *d*log *y*_0_/*d*(1 - *q*) is locally minimum as depicted in (C). For all graphs, the parameters are as follows. *ν *= 50. *f*_*d *_= 0.99^*d*^. *κ *= 0.05; however, for each G
 MathType@MTEF@5@5@+=feaafiart1ev1aaatCvAUfKttLearuWrP9MDH5MBPbIqV92AaeXatLxBI9gBaebbnrfifHhDYfgasaacH8akY=wiFfYdH8Gipec8Eeeu0xXdbba9frFj0=OqFfea0dXdd9vqai=hGuQ8kuc9pgc9s8qqaq=dirpe0xb9q8qiLsFr0=vr0=vr0dc8meaabaqaciaacaGaaeqabaqabeGadaaakeaat0uy0HwzTfgDPnwy1egaryqtHrhAL1wy0L2yHvdaiqaacqWFge=raaa@382C@_*d*_, Round(κ(νd))/(νd)
 MathType@MTEF@5@5@+=feaafiart1ev1aaatCvAUfKttLearuWrP9MDH5MBPbIqV92AaeXatLxBI9gBaebbnrfifHhDYfgasaacH8akY=wiFfYdH8Gipec8Eeeu0xXdbba9frFj0=OqFfea0dXdd9vqai=hGuQ8kuc9pgc9s8qqaq=dirpe0xb9q8qiLsFr0=vr0=vr0dc8meaabaqaciaacaGaaeqabaqabeGadaaakeaadaWcgaqaamaabmaabaacciGae8NUdS2aaeWaaeaafaqabeGabaaabaGae8xVd4gabaGaemizaqgaaaGaayjkaiaawMcaaaGaayjkaiaawMcaaaqaamaabmaabaqbaeqabiqaaaqaaiab=17aUbqaaiabdsgaKbaaaiaawIcacaGLPaaaaaaaaa@3938@ is used as the actual value of *κ *in Eq. (2), where Round(*x*) is the closest integer of *x*. (Thus, *κ *= 0 for G
 MathType@MTEF@5@5@+=feaafiart1ev1aaatCvAUfKttLearuWrP9MDH5MBPbIqV92AaeXatLxBI9gBaebbnrfifHhDYfgasaacH8akY=wiFfYdH8Gipec8Eeeu0xXdbba9frFj0=OqFfea0dXdd9vqai=hGuQ8kuc9pgc9s8qqaq=dirpe0xb9q8qiLsFr0=vr0=vr0dc8meaabaqaciaacaGaaeqabaqabeGadaaakeaat0uy0HwzTfgDPnwy1egaryqtHrhAL1wy0L2yHvdaiqaacqWFge=raaa@382C@_*ν*_, and then *f*_*ν *_is set to zero to be consistent).

The stationary solution of Eq. (2) can be calculated as a normalized eigen vector associated with the greatest eigen value of the matrix representing the linear part of Eq. (2) [15,16]. First, the stationary solution was calculated without the lethality of mutants as depicted in Fig. 2 (the analytical solution is known [9]). As Fig. 2 shows, the population fraction of every genotype rapidly and smoothly converges to the same limiting value, which is the population fraction for completely random replication (i.e., the population fraction when 1 - *q *= 0.5). [The above observation does not differ qualitatively when the fitness landscape is steeper (say *f*_*d *_= 0.9^*d*^), or when sequence length is different – the convergence is faster when sequence length is longer (see below)]. Thus, it can be said that the population is delocalized from the fittest genotype for error rates sufficiently high but yet much smaller than that of completely random replication – evolutionary optimization breaks down. Furthermore, the population distribution does not show any discontinuous transition, and thus there is no error "threshold" as already stated in [9]. The next step of the study is to examine the effect of the lethality of mutants on the behavior of Eq. (2). The stationary solutions of Eq. (2) were numerically calculated with lethality. A typical result of calculation is depicted in Fig. 3 for high lethality [*κ *= 0.05; however, note that Round(κ(νd))/(νd)
 MathType@MTEF@5@5@+=feaafiart1ev1aaatCvAUfKttLearuWrP9MDH5MBPbIqV92AaeXatLxBI9gBaebbnrfifHhDYfgasaacH8akY=wiFfYdH8Gipec8Eeeu0xXdbba9frFj0=OqFfea0dXdd9vqai=hGuQ8kuc9pgc9s8qqaq=dirpe0xb9q8qiLsFr0=vr0=vr0dc8meaabaqaciaacaGaaeqabaqabeGadaaakeaadaWcgaqaamaabmaabaacciGae8NUdS2aaeWaaeaafaqabeGabaaabaGae8xVd4gabaGaemizaqgaaaGaayjkaiaawMcaaaGaayjkaiaawMcaaaqaamaabmaabaqbaeqabiqaaaqaaiab=17aUbqaaiabdsgaKbaaaiaawIcacaGLPaaaaaaaaa@3938@ is used as the actual value of *κ *for each G
 MathType@MTEF@5@5@+=feaafiart1ev1aaatCvAUfKttLearuWrP9MDH5MBPbIqV92AaeXatLxBI9gBaebbnrfifHhDYfgasaacH8akY=wiFfYdH8Gipec8Eeeu0xXdbba9frFj0=OqFfea0dXdd9vqai=hGuQ8kuc9pgc9s8qqaq=dirpe0xb9q8qiLsFr0=vr0=vr0dc8meaabaqaciaacaGaaeqabaqabeGadaaakeaat0uy0HwzTfgDPnwy1egaryqtHrhAL1wy0L2yHvdaiqaacqWFge=raaa@382C@_*d *_in Eq. (2), where Round(*x*) is the closest integer of *x*. In the following this is true unless otherwise indicated]. As Fig. 3 shows, the population fraction of the fittest genotype shows a sharp transition at some critical error rate in contrast to the case without lethality, and the population distribution suddenly converges to the same value around this transition. [The above observations do not differ qualitatively when the fitness landscape is steeper (say *f*_*d *_= 0.9^*d*^) or when sequence length is different – the transition is sharper when sequence length is longer (see below).] Therefore, the lethality of mutants can actually introduce an error-threshold in sharp contrast to the conclusion of [5,8], and furthermore, even with high lethality of mutants the population can still be delocalized from the fittest genotype although the error rate for this to happen is higher than the case without lethality.

Below, we study the emergence of an error-threshold due to the lethality of mutants and the delocalization of population in more detail.

#### How high must lethality be to introduce an error-threshold?

**Figure 4:**
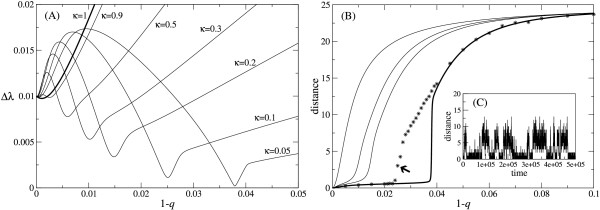
**Identification of the error-thresholds**. **(A) **The difference between the greatest and the second greatest eigen value (Δ*λ*) is plotted against the error rate (1 - *q*). The error rate for which Δ*λ *is minimum can be identified as the error-threshold, *ν *= 50 and *f*_*d *_= 0.99^*d*^. The value of *κ *is indicated in the graph. [The actual value of *κ *is determined in the same way as in Fig. 3, and this is true in this figure unless otherwise stated.] For *κ *= 1, the line is thicker. **(B) **The average Hamming distance of the ancestor distribution from the fittest genotype, ∑d=0νad(∞,∞)
 MathType@MTEF@5@5@+=feaafiart1ev1aaatCvAUfKttLearuWrP9MDH5MBPbIqV92AaeXatLxBI9gBaebbnrfifHhDYfgasaacH8akY=wiFfYdH8Gipec8Eeeu0xXdbba9frFj0=OqFfea0dXdd9vqai=hGuQ8kuc9pgc9s8qqaq=dirpe0xb9q8qiLsFr0=vr0=vr0dc8meaabaqaciaacaGaaeqabaqabeGadaaakeaadaaeWaqaaiabdggaHnaaBaaaleaacqWGKbazaeqaaOWaaeWaaeaacqGHEisPcqGGSaalcqGHEisPaiaawIcacaGLPaaaaSqaaiabdsgaKjabg2da9iabicdaWaqaaGGaciab=17aUbqdcqGHris5aaaa@3BCF@, is plotted against 1 - *q*. The figure depicts the genealogical delocalization of the population from the fittest genotype for high error rates irrespective of the lethality of mutants, and the clear existence of the error-threshold for high lethality of mutants. The thick solid line is for *κ *= 0.05. The other solid lines are for *κ *= 1, 0.3, 0.2, respectively from left to right. **The stars **represent the average Hamming distance between the common ancestors and the fittest genotype obtained from the finite population model (see text). Note that the ancestors from the early simulation (< 10000) steps were discarded to consider the system only at an equilibrium. *κ *= 0.05. **The arrow **represents a simulation run which is depicted in (C). **(C) **The Hamming distance of the common ancestors is plotted against the time step at which the common ancestors were born. The meta-stability is observed as random switching between two modes. The plot was obtained from the simulation run indicated by the arrow in (B). 1 - *q *= 0.025.

As seen above, high lethality of mutants can, in fact, introduce an error-threshold. In the following, we elucidate the relationship between lethality and the error-threshold by extending the characterization of the error-threshold. The method employed for this sake, which was introduced in [17], is as follows. The difference between the greatest eigen value and the second greatest eigen value of the linear part of Eq. (2) becomes minimum at the error-threshold, and hence, this provides a convenient measure of the error-threshold. As Fig. 4(A) shows, there is an error rate for which the greatest eigen value becomes very close to the second greatest eigen value for a sufficiently small value of *κ *and this error rate is identified as the error-threshold. However, for a greater *κ*, the difference between the two eigen values does not become very small, and this corresponds to the absence of the error-threshold for a high value of *κ*. However, it is noteworthy that the characteristic shape of the curves in Fig. 4(A) is present for all *κ *< 1 shown in the figure, and therefore it is possible to identify a distinctive error rate for which the difference between the eigen values is minimum.

#### Effect of sequence length on the error-threshold

To examine the effect of sequence length on the error-threshold, the error-threshold measured through the above method is plotted for various sequence length in Fig. 1(A) (filled circles) for high lethality of mutants (*κ *= 0.05). As is clear from this plot, the error-threshold decreases as *ν *increases. Furthermore, as shown by Fig. 1(B) (circles), the maximum tolerable genomic mutation rate [i.e., (1 - *q*_min_)*ν*] increases as *ν *increases, which strengthen the last result [see also Fig. 1(C) in terms of *f*_0_/*f*_1_]. As discussed previously, the increase of (1 - *q*_min_)*ν *is due to the assumption of a constant *f*_0_/*f*_1 _with respect to *ν*, under which *f*_0_/fν2
 MathType@MTEF@5@5@+=feaafiart1ev1aaatCvAUfKttLearuWrP9MDH5MBPbIqV92AaeXatLxBI9gBaebbnrfifHhDYfgasaacH8akY=wiFfYdH8Gipec8Eeeu0xXdbba9frFj0=OqFfea0dXdd9vqai=hGuQ8kuc9pgc9s8qqaq=dirpe0xb9q8qiLsFr0=vr0=vr0dc8meaabaqaciaacaGaaeqabaqabeGadaaakeaacqWGMbGzdaWgaaWcbaWaaSaaaeaaiiGacqWF9oGBaeaacqaIYaGmaaaabeaaaaa@30EE@ increases exponentially as *ν *increases. If *f*_0_/fν2
 MathType@MTEF@5@5@+=feaafiart1ev1aaatCvAUfKttLearuWrP9MDH5MBPbIqV92AaeXatLxBI9gBaebbnrfifHhDYfgasaacH8akY=wiFfYdH8Gipec8Eeeu0xXdbba9frFj0=OqFfea0dXdd9vqai=hGuQ8kuc9pgc9s8qqaq=dirpe0xb9q8qiLsFr0=vr0=vr0dc8meaabaqaciaacaGaaeqabaqabeGadaaakeaacqWGMbGzdaWgaaWcbaWaaSaaaeaaiiGacqWF9oGBaeaacqaIYaGmaaaabeaaaaa@30EE@ is kept constant, (1 - *q*_min_)*ν *actually decreases and then saturates as *ν *increases as shown in Fig. 1(B), which is comparable to the result of [3,14] [see also Fig. 1(D)]. Thus, these results are in concordance with the results from Eq. (4). In conclusion, the error-threshold depends negatively on *ν *even under the assumption of constant *f*_0_/*f*_1_.

#### Effect of lethality on the ancestor distribution

Above, the effect of lethality on the mutation-selection dynamics was studied in terms of the equilibrium population distribution. From the evolutionary point of view, however, it will add significant information to study this in terms of the genealogical success of each genotype in an evolutionary time scale. The "ancestor distribution" [18] reveals which ancestral genotype (or genotype class) the current population has originated from. By following [18], the ancestor distribution is defined as ad(∞,∞)≡lim⁡τ→∞t→∞ad(τ,t)
 MathType@MTEF@5@5@+=feaafiart1ev1aaatCvAUfKttLearuWrP9MDH5MBPbIqV92AaeXatLxBI9gBaebbnrfifHhDYfgasaacH8akY=wiFfYdH8Gipec8Eeeu0xXdbba9frFj0=OqFfea0dXdd9vqai=hGuQ8kuc9pgc9s8qqaq=dirpe0xb9q8qiLsFr0=vr0=vr0dc8meaabaqaciaacaGaaeqabaqabeGadaaakeaacqWGHbqydaWgaaWcbaGaemizaqgabeaakmaabmaabaGaeyOhIuQaeiilaWIaeyOhIukacaGLOaGaayzkaaGaeyyyIORagiiBaWMaeiyAaKMaeiyBa02aa0baaSqaaGGaciab=r8a0jabgkziUkabg6HiLcqaaiabdsha0jabgkziUkabg6HiLcaakiabdggaHnaaBaaaleaacqWGKbazaeqaaOWaaeWaaeaacqWFepaDcqGGSaalcqWG0baDaiaawIcacaGLPaaaaaa@4D4C@, where *a*_*d*_(*τ*, *t*) is the fraction of the population at time *t *+ *τ *(*τ *> 0) of which lineage is traced back to the population of the genotype class G
 MathType@MTEF@5@5@+=feaafiart1ev1aaatCvAUfKttLearuWrP9MDH5MBPbIqV92AaeXatLxBI9gBaebbnrfifHhDYfgasaacH8akY=wiFfYdH8Gipec8Eeeu0xXdbba9frFj0=OqFfea0dXdd9vqai=hGuQ8kuc9pgc9s8qqaq=dirpe0xb9q8qiLsFr0=vr0=vr0dc8meaabaqaciaacaGaaeqabaqabeGadaaakeaat0uy0HwzTfgDPnwy1egaryqtHrhAL1wy0L2yHvdaiqaacqWFge=raaa@382C@_*d *_present at time *t*. The calculation of *a*_*d*_(∞, ∞) is explained in [18] in detail (see also [19,20] for the treatment of the same concept by statistical mechanics).

The average Hamming distance of the ancestor distribution from the fittest genotype, ∑d=0νad(∞,∞)
 MathType@MTEF@5@5@+=feaafiart1ev1aaatCvAUfKttLearuWrP9MDH5MBPbIqV92AaeXatLxBI9gBaebbnrfifHhDYfgasaacH8akY=wiFfYdH8Gipec8Eeeu0xXdbba9frFj0=OqFfea0dXdd9vqai=hGuQ8kuc9pgc9s8qqaq=dirpe0xb9q8qiLsFr0=vr0=vr0dc8meaabaqaciaacaGaaeqabaqabeGadaaakeaadaaeWaqaaiabdggaHnaaBaaaleaacqWGKbazaeqaaOWaaeWaaeaacqGHEisPcqGGSaalcqGHEisPaiaawIcacaGLPaaaaSqaaiabdsgaKjabg2da9iabicdaWaqaaGGaciab=17aUbqdcqGHris5aaaa@3BCF@, is plotted for various values of *κ *as a function of error rate in Fig. 4(B) (solid lines). As shown in this plot, the average distance of the ancestors is close to zero for a sufficiently small error rate; that is, after a sufficiently long time almost the entire population has originated from the fittest genotype – evolutionary optimization is effective. However, as error rate increases, the average distance of the ancestors rapidly approaches to a half of the sequence length (50/2 in the current setting) irrespective of the degree of lethality (1 - *κ*). This means that for a sufficiently high error rate, the population has mostly originated from near random sequences – evolutionary optimization breaks down. In conclusion, the ancestor distribution clearly illustrates the genealogical delocalization of the population at a sufficiently high error rate irrespective of the lethality of mutants, as is consistent with the results from the equilibrium population distribution. Furthermore, as seen in the same plot [Fig. 4(B)], for high lethality of mutants (a small value of *κ*), there is clearly a sharp transition in the average distance of the ancestors, which identifies the error-threshold. In conclusion, as is consistent with the results from the equilibrium population distribution, the ancestor distribution shows the existence of an error-threshold for high lethality of mutants in a multiplicative fitness landscape. In fact, the error-threshold is sharper in the ancestor distribution [20].

#### Effect of finite populations

To examine the above results without the assumption of infinite population size, we study the role of lethality (with back mutations) in relatively small finite populations by focusing on the the ancestors of the population (see [17,21,22], for the study of the error-threshold in finite populations in terms of the equilibrium population distribution). However, in the system of a finite population, the entire population will share a single last common ancestor after a sufficiently long time in contrast to the system of infinite population. Thus, instead of the ancestor distribution, here we study the distribution of the common ancestors of the population (the last common ancestor and its ancestors).

We constructed a finite population model which follows mutation-selection dynamics described by Eq. (2), by basically following the implementation described in [13]. In the current model, lethality was implemented as discarding a new born individual which is determined as a lethal mutant. For simplicity, determining a new born as a lethal mutant was implemented as a chance event with the probability of 1 - *κ*; thus, genotypes were not explicitly assigned as lethal genotypes. The population size was approximately 10^5^, and *ν *= 50 (thus, the population size is far smaller than the number of possible sequences), and *f*_*d *_= 0.9 × 0.99^*d *^(the probability of replication per time step).

In the above model, the average Hamming distance between the fittest genotype and the common ancestors was calculated after sufficiently long simulation steps (5 × 10^5 ^steps). In Fig. 4(B) (dots), a typical result of such a calculation is plotted for high lethality of mutants (*κ *= 0.05) as a function of error rate. As seen from this plot, the distance of the common ancestors matches very well with the distance of the ancestor distribution from the infinite population model [Fig. 4(B), the thick solid line] for small and large error rates, exhibiting the genealogical delocalization of the population for high error rates. Moreover, as seen in the same plot, the distance of the ancestor distribution suddenly increases at a critical error-rate, which indicates the existence of a error-threshold. This error-threshold is smaller than that of the infinite population as expected [17,21,22]. We next take a closer look at the finite population model for error rates between the error-threshold of the infinite population model and that of the finite population model. In Fig. 4(C), the distance of the common ancestors obtained from the finite population model is plotted against the time step at which the common ancestors were born [the error rate chosen for this plot is indicated by the arrow in Fig. 4(B)]. As seen from this plot, there are two meta-stable modes in the common ancestor distribution. One mode corresponds to the ancestor distribution of the infinite population model below the error-threshold (ordered mode); the other corresponds to that above the error-threshold (disordered mode). Apparently, due to the stochasticity and the proximity of the two greatest eigen values [see Fig. 4(A)], the common ancestor distribution randomly switches between the two modes. [When the error rate is close to the error-threshold of the infinite population model, the system shows almost always the disordered mode.] The above results show that the system of a finite population also displays a transition-like behavior in the distribution of the common ancestors although in a qualitatively different manner from that of the ancestor distribution of the infinite population model. In conclusion, the error-threshold exists for high lethality of mutants also in the finite population model.

## Discussion

In the current study, high-dimensional genotype space and multiple substitutions per replication were taken into consideration, in order to investigate the effect of mutant lethality on mutation-selection dynamics. On one hand, the model was analyzed by neglecting back mutations to compare the results with [8]. The results showed the extinction of the fittest genotype can happen even with high lethality of mutants because of mutations within the same genotype class. On the other hand, the model was analyzed with the consideration of back mutations from three aspects. The first aspect is the equilibrium population distribution, which tells the state of the population at one moment after a sufficiently long time. The second aspect is the difference between the greatest and second greatest eigen values, which represents the aspect of singularity in the population distribution as a function of error rate. The last aspect is the ancestor distribution. This tells the genealogical description of the population in a long time scale, which is significant information from the point of view of evolution. The results showed that the investigations from these three aspects give a consistent picture about the error-threshold introduced by high lethality of mutants and about the delocalization of the population from the fittest genotype for a sufficiently high error rate irrespective of the lethality of mutants. Furthermore, the effect of a finite population was studied, and our results were shown to extend to the system of finite populations.

Four additional points should be noted in the current study. First, the biological implication of the error-threshold is that it hampers the accumulation of information through increasing the genome size of the replicators [1,4]. In the case of low lethality of mutants, it was shown that there is no error-threshold in a multiplicative fitness landscape. However, this does not mean that the accumulation of information is not hampered; on the contrary, it is hampered by the delocalization of the population from the fittest genotype. Second, the population size was assumed to be constant as is often done. An artifact of this simplification is, among others, the impossibility of population extinction. Third, the fitness was assumed to be a function of the number of substitutions (*d*) so that the grouping of genotypes by *d *is straightforward. For other settings, one can consult [23], which estimates the error-threshold in a fitness landscape where the fitness of a genotype is determined from a probability distribution. Forth, the error-threshold was considered only in terms of the fittest replicator. However, the error-threshold can be also considered for mutant classes (e.g. [22,24]). As shown in [22] for "Royal Road fitness landscape", if the error rate is beyond the error-threshold of the fittest, the population can still be localized in sequence space albeit at a lower fitness level, for which a new error-threshold again exists.

## Conclusion

• Irrespective of the lethality of mutants, the delocalization of the population from the fittest genotype – the break down of evolutionary optimization – happens for an error rate much smaller than that of random replication.

• High lethality of mutants introduces an error-threshold in a multiplicative fitness landscape.

## Authors' contributions

NT is the main author. PH supervised NT in all aspects. All authors read and approved the final manuscript.

## Response

By Claus O Wilke

E-Mail: cwilke@mail.utexas.edu

Address: Section of Integrative Biology, Center for Computational Biology and Bioinformatics, and Institute for Cell and Molecular Biology, University of Texas at Austin, Austin, Texas 78712, USA

Quasispecies theory [25-27] is a deterministic theory of mutation-selection balance for multi-site models, applicable in particular but not exclusively to settings of high mutation pressure, such as the evolution of RNA viruses. The theory has often been perceived as an alternative to and possibly even contradictory to classical population genetics. In a recent review [28], I tried to clear up these misconceptions, and demonstrated that the quasispecies equations are equivalent to the equations used in population genetics to describe the deterministic evolution of asexual replicators. I also touched on the error threshold, probably the most widely discussed and at the same time most frequently misunderstood aspect of quasispecies theory. I argued that the existence and location of the error threshold are not universal, and that the error threshold is not related to population extinction. Moreover, citing a proof by Wagner and Krall [29], I claimed that an error threshold cannot occur if a fitness landscape contains lethal genotypes.

Takeuchi and Hogeweg [30] now present a counterexample to my claim, a fitness landscape on which an error threshold occurs even though lethal genotypes exist. This finding does not invalidate Wagner and Krall's work, but it demonstrates that we cannot generalize Wagner and Krall's result – derived for a one-dimensional fitness landscape – to higher dimensions. Takeuchi and Hogeweg's result is a welcome contribution to the quasispecies literature, and it reminds us that generalization from simplified mathematical models to more complex situations can be dangerous. However, this result is of minor consequence to my review. The main conclusions in my the section on error thresholds remain valid: (i) Whether an error threshold exists depends on the details of the fitness landscape. For example, as Takeuchi and Hogeweg acknowledge, the purely multiplicative fitness landscape does not display an error threshold. (ii) If an error threshold exists, its location depends on the details of the fitness landscape as well. This fact is demonstrated for example in Fig. 1 of Takeuchi and Hogeweg's article. (iii) Most importantly, the error threshold is not related to publication extinction. Population extinction occurs when the absolute population size *N *drops to zero. Models of the error threshold, including the one by Takeuchi and Hogeweg, generally operate on *relative *mutant frequencies while holding the total population size *N *constant. If we generalize the quasispecies equations to allow for variable *N*, we find – in the deterministic limit, i.e., for large *N *– that population extinction is not tied to the error threshold [31,32]. In fact, under certain conditions, an error threshold may delay or even prevent population extinction brought about by an increase in the mutation rate [32].

To summarize, while Takeuchi and Hogeweg make a valid and important contribution to our understanding of error thresholds, their work does not affect my conclusions on the relationship between quasispecies theory and population genetics, nor does it invalidate in any substantive way the statements I made about error thresholds.

### Acknowledgements

C.O.W. is funded by NIH grant AI 065960.
